# BLINCAR: a reusable bioluminescent and Cas9-based genetic toolset for repeatedly modifying wild-type *Scheffersomyces stipitis*

**DOI:** 10.1128/msphere.00224-23

**Published:** 2023-06-22

**Authors:** Walter D. Reichard, Serenah E. Smith, J. Brian Robertson

**Affiliations:** 1 Department of Biology, Middle Tennessee State University, Murfreesboro, Tennessee, USA; University of Georgia, Athens, Georgia, USA

**Keywords:** bioluminescence, Cas9, *S. stipitis*, CUG group yeast

## Abstract

*Scheffersomyces stipitis* is a yeast that robustly ferments the 5-carbon sugar xylose, making the yeast a valuable candidate for lignocellulosic ethanol fermentation. However, the non-canonical codon usage of *S. stipitis* is an obstacle for implementing molecular tools that were developed for other yeast species, thereby limiting the molecular toolset available for *S. stipitis*. Here, we developed a series of molecular tools for *S. stipitis* including BLINCAR, a Bio-Luminescent Indicator that is Nullified by Cas9-Actuated Recombination, which can be used repeatedly to add different exogenous DNA payloads to the wild-type *S. stipitis* genome or used repeatedly to remove multiple native *S. stipitis* genes from the wild-type genome. Through the use of BLINCAR tools, one first produces antibiotic-resistant, bioluminescent colonies of *S. stipitis* whose bioluminescence highlights those clones that have been genetically modified; then second, once candidate clones have been confirmed, one uses a transient Cas9-producing plasmid to nullify the antibiotic resistance and bioluminescent markers from the prior introduction, thereby producing non-bioluminescent colonies that highlight those clones which have been re-sensitized to the antibiotic and are therefore susceptible to another round of BLINCAR implementation.

**IMPORTANCE:**

Cellulose and hemicellulose that comprise a large portion of sawdust, leaves, and grass can be valuable sources of fermentable sugars for ethanol production. However, some of the sugars liberated from hemicellulose (like xylose) are not easily fermented using conventional glucose-fermenting yeast like *Saccharomyces cerevisiae*, so engineering robust xylose-fermenting yeast that is not inhibited by other components liberated from cellulose/hemicellulose will be important for maximizing yield and making lignocellulosic ethanol fermentation cost efficient. The yeast *Scheffersomyces stipitis* is one such yeast that can ferment xylose; however, it possesses several barriers to genetic manipulation. It is difficult to transform, has only a few antibiotic resistance markers, and uses an alternative genetic code from most other organisms. We developed a genetic toolset for *S. stipitis* that lowers these barriers and allows a user to deliver and/or delete multiple genetic elements to/from the wild-type genome, thereby expanding *S. stipitis’s* potential.

## INTRODUCTION

The need for a renewable, carbon-neutral liquid fuel has energized the field of lignocellulosic ethanol production research. Developing technologies that allow yeasts to efficiently ferment the sugars found in wood, leaves, and agricultural stover into ethanol in a cost-effective manner may provide alternatives to gasoline and diesel (1). Although *Saccharomyces cerevisiae* is one of the most researched yeasts for ethanol fermentation and has an extensive set of molecular tools that have been developed for manipulating its genome and biochemical pathways, another yeast, *Scheffersomyces stipitis,* is gaining popularity due to its active pentose metabolism pathways and ability to ferment the xylose sugar found as a major component of lignocellulosic biomass (2). However, *S. stipitis* growth and xylose fermentation are particularly sensitive to even low levels of ethanol (3) and other sugars liberated from lignocellulose (4), so engineering alcohol tolerance and glucose insensitivity for these pathways will require extensive genetic manipulation and a diverse set of molecular tools for *S. stipitis* (5).

Wild-type *S. stipitis* has several obstacles preventing manipulation of its genome and biochemical pathways. In addition to its general inefficiency of transformation (6, 7), the chief obstacle is that *S. stipitis* belongs to a paraphyletic group of yeast (including *Candida albicans*) in which the codon CUG translates to the amino acid serine instead of leucine like it does for most other organisms (8). This alternative codon usage means that most genetically encoded molecular tools and heterologous proteins developed for use in other organisms (including other yeast such as *S. cerevisiae*) do not function in *S. stipitis* without first codon-optimizing their DNA sequences to account for the alternative CUG codon assignment. Because of this, genetically encoded molecular tools like reporter genes, selectable markers, and Cas9 technologies for *S. stipitis* are limited.

Others have worked to expand the molecular toolset, but in many cases, these tools come with limitations that must be weighed. For instance, a fluorescent reporter gene (enhanced green fluorescent protein) has been used to measure promoter activity for suspected promoters in *S. stipitis* (9), but unless cells are strongly overexpressing a fluorescent protein, it is difficult to use fluorescent reporters in colonies on agar plates and in rich media due to high levels of autofluorescence caused by the medium and agar (10). Others have created auxotrophic strains of *S. stipitis* with selectable markers that bypass the auxotrophy (7, 11, 12); however, these selectable markers do not work in a wild-type strain of *S. stipitis*, and obtaining these auxotrophic strains can be problematic if their creator does not wish to share them. And although others have built CRISPR/Cas9 tools for *S. stipitis* (13), the wild-type strain’s prevalence for quickly repairing double-stranded breaks by non-homologous end joining (NHEJ) rather than homologous recombination (HR) makes Cas9-based gene modification difficult, requiring instead special strains of *S. stipitis* that have been engineered to reduce their prevalence for NHEJ rather than using wild-type yeast (14).

In this work, we describe the development and implementation of BLINCAR (Bio Luminescent Indicator Nullified by Cas-9 Actuated Recombination), a reusable CRISPR/Cas9-based bioluminescent detectable and selectable reporter system for delivering and/or removing genetic elements to/from the wild-type *S. stipitis* genome and reestablishing sensitivities to the antibiotics used in its implementation. BLINCAR follows a similar approach to a previously reported LoxP/Cre-based system developed for re-sensitizing *S. stipitis* to selection strategies after their initial use (15). But our technology improves on this approach by (i) using Cas9 to target an artificial motif unique to the BLINCAR element instead of Cre-Lox sites which are inherently problematic in PCR due to the LoxP sequence causing hairpins in primers, (ii) including a bioluminescent reporter gene that allows efficient screening of colonies on rich media for the addition or removal of the BLINCAR element, and (iii) uses antibiotic selection strategies as opposed to auxotrophic ones to allow the system to work in wild-type strains of *S. stipitis*. Through its construction, we also developed several supporting codon-optimized genetic tools for *S. stipitis* (and other related yeast), including a vector with an extensive multiple cloning site (over 50 unique restriction sites), a bioluminescent reporter for *in vivo* candidate promoter evaluation, an expanded set of antibiotic resistance genes, and a single plasmid-based Cas9/sgRNA delivery system. We demonstrate how BLINCAR can be used to deliver multiple genetic payloads (e.g., heterologous genes) to the *S. stipitis* genome as well as remove targeted native *S. stipitis* genes from the genome.

## MATERIALS AND METHODS

### Strains, media, and growth conditions

TOP10 chemically competent *Escherichia coli* cells (Invitrogen, Carlsbad, CA) were used for all plasmid maintenance and cloning. Wild-type *S. stipitis* strain CBS 6054 (ATCC 58785) was used for all yeast experiments. *E. coli* were grown in solid or liquid LB (Luria-Bertani) medium containing 100 µg/mL ampicillin overnight. *S. stipitis* was grown overnight at 30°C in liquid YPD (1% yeast extract, 2% peptone, and 2% dextrose) or on solid YPD (2% agar) with or without one of the following antibiotics: 100 µg/mL Zeocin (Invitrogen, Carlsbad, CA), 200 µg/mL hygromycin B (RPI, Mt. Prospect, IL), 10–100 µg/mL Nourseothricin (RPI), or 200 µg/mL G-418 (RPI). For some *ura3* deletion experiments, *S. stipitis* was grown on synthetic minimal medium without uracil (6.7 g/L Difco yeast nitrogen base without amino acids, 770 mg/L, Formedium CSM drop-out mix without uracil, 2% glucose, and 2% agar). For other experiments, this minimal medium was supplemented with 100 µg/mL of uracil.

### Bioluminescence gene selection

The gene for click beetle green luciferase “CBG99” (Promega, Madison, WI) was chosen as the bioluminescence reporter gene for this work because the protein’s green emission was robust when produced by yeast on rich, solid media and can be visually distinguished from colonies producing red-emitting luciferases should the need for dual-color assays arise (16). A codon-optimized version of Promega’s CBG99 gene was developed for expression by *S. stipitis* and called coCBG for this work.

### Plasmid construction

All plasmids (see Table 1; Table S1) were constructed using a traditional restriction enzyme-based cloning approach. See supplement for a full description of construction methods; but briefly, heterologous sections of DNA (inserts) were either cut directly out of synthesized plasmids or amplified from *S. stipitis* genomic DNA or template plasmids by PCR using primers (see Table 2) that added specific restriction sites to the ends of the PCR product. Once a heterologous segment (insert) and plasmid vector were digested with the appropriate restriction enzyme(s), they were run on 1% agarose gel and bands of appropriate sizes were isolated, DNA purified, and ligated together. Ligation products were transformed into competent TOP10 *E. coli*. Each piece of the constructed plasmid was sequentially added in this way.

**TABLE 1:** Plasmids

Plasmid name	Features and notes
	**Synthesized plasmids from this work**
pAllet	pUC57 with synthesized large MCS produced by GenScript
pUC57-coHPH	pUC57 with synthesized coHPH produced by GenScript
pUC57-coKanMX	pUC57 with synthesized coKanMX produced by GenScript
pUC57-coNat	pUC57 with synthesized coNat produced by GenScript
pUC57-coShBle	pUC57 with synthesized coShBle produced by GenScript
pUC57-coCBG	pUC57 with synthesized coCBG produced by GenScript
pUC57-sgRNAsap	pUC57 with synthesized tRNA-Ala, SapI stuffer, tracrRNA, and HDV element produced by GenScript
	**Reporter plasmids from this work**
pWR9	pAllet with P_TEF1_-coHPH, P_TDH3_-coCBG, ARS-1 (episome)
pWR10	pAllet with P_TEF1_-coShBle, P_TDH3_-coCBG, ARS-1 (episome)
pWR11	pAllet with P_TEF1_-coHPH, P_GAL1_-coCBG, ARS-1 (episome)
pWR16	pAllet with P_TEF1_-coHPH, P_TDH3_-coCBG, *URA3* (integration)
pWR22	pAllet with P_TEF1_-coHPH, P_XKS1_-coCBG, ARS-1 (episome)
pWR23	pAllet with P_TEF1_-coKanMX, P_TDH3_-coCBG, ARS-1 (episome)
pWR24	pAllet with P_TEF1_-coNat, P_TDH3_-coCBG, ARS-1 (episome)
pWR54	pAllet with P_TEF1_-coHPH, P_CCW12_-coCBG, ARS-1 (episome)
pWR55	pAllet with P_TEF1_-coHPH, P_PGK1_-coCBG, ARS-1 (episome)
pWR56	pAllet with P_TEF1_-coHPH, P_HHF1_-coCBG, ARS-1 (episome)
pWR76	pAllet with P_TEF1_-coHPH, P_TDH3_-coCherry, ARS-1 (episome)
pWR77	pAllet with P_TEF1_-coHPH, P_TEF1_-coCBG, ARS-1 (episome)
pWR78	pAllet with P_TEF1_-coHPH, P_ADH1_-coCBG, ARS-1 (episome)
pWR81	pAllet with P_TEF1_-coHPH, P_CYC1_-coCBG, ARS-1 (episome)
pWR84	pAllet with P_TEF1_-coHPH, P_TDH3_-coCBG, *EGC3* (integration)
pWR85	pAllet with P_TEF1_-coHPH, P_TDH3_-coCBG, *GSC2* (integration)
pWR86	pAllet with P_TEF1_-coHPH, P_TDH3_-coCBG, *CRF1* (integration)
	**CAS9-related/BLINCAR-related plasmids from this work**
pWR58	pAllet with P_TEF1_-coShBle, P_TDH3_-interupted coCBG, *URA3* (integration)
pWR63	pAllet with P_TEF1_-coHPH, P_CCW12_-CaCas9, P_TDH3_-NOsgRNA, ARS-1 (episome)
pWR63(YUM1)	pAllet with P_TEF1_-coHPH, P_CCW12_-CaCas9, P_TDH3_-YUM1sgRNA, ARS-1
pWR88	BLINCAR for payload delivery: no payload, no target
pWR89	BLINCAR for payload delivery: no payload, *EGC3* (integration)
pWR90	BLINCAR for payload delivery: no payload, *GSC2* (integration)
pWR91	BLINCAR for payload delivery: no payload, *CRF1* (integration)
pWR95	BLINCAR for gene deletion: no target
pWR95(URA3)	BLINCAR for gene deletion: *URA3* target
pWR96	pAllet with P_TEF1_-coHPH, P_TDH3_-coCBG, *URA3*, ARS-1 (episome)
pWR101	BLINCAR for payload delivery: P_CCW12_-coCherry, *EGC3* (integration)
pWR102	BLINCAR for payload delivery: P_CCW12_-coCherry, *GSC2* (integration)
pWR103	BLINCAR for payload delivery: P_CCW12_-coCherry, *CRF1* (integration)
	**Plasmids from others**
yEpGAP-Cherry	Contains red fluorescent protein gene (coCherry) optimized for *Candida albicans* from reference (17)
pV1093	Contains Cas9 gene codon optimized for *Candida albicans* from reference (18)
pUC8-*lacZ*	Plasmid containing *E. coli lacZ* gene from Elliot Altman, MTSU

**TABLE 2:** Primers

No.	Primer name	Sequence (5′-3′)^*a*^
1	Ptef(Xho)5	tgactg**CTCGAG** CCGTACACTTATTGTTAACTATGAA
2	Ptef(Brsg)3	tgccat**TGTACA** TGTAGATAGACTTAGATTGTATGAAA
3	ACT1term(Not)5	tgacgt**GCGGCCGC**tAACCACTTGCAAAATCCTTTG
4	ACT1term(Sal)3	tgccat**GTCGAC** GAACTTGATTGTAATACAAAATGC
5	Ptdh3(Bam)5	tcatta**GGATCC** TCGTTAGTATTTCCGTGAAG
6	Ptdh3(Kpn)3	atgact**GGTACC** GATGAATTGTTTATAGGGAAGA
7	Adh2term(Afl)5	atactg**CTTAAG** CATTTTAGACAAGTGCCTATT
8	Adh2term(EcoRI)3	tgccat**GAATTC** TTCTGCCTTCTGAACGTTTG
9	PsARS5(Sacl)	aacttg**GAGCTC** AGTATAGGATATGGTGATTTAGC
10	PsARS3(Sacl)	caagtt**GAGCTC** TCTGCGGTGTCTACAAGGTC
11	UraUS(SacI)5	acttcc**GAGCTC** AGTACGAACAAGAGAATGA
12	UraDS(SacI)3	acttcc**GAGCTC** ACAGGGTTGATATTGTACG
13	Pgal1(Bam)5	attcca**GGATCC** GAAGAACTTGTTCTGAAAGC
14	Pgal1(Kpn)3	acttca**GGTACC** AGTAATAGAAACAACAGAACGT
15	Pxks1(Bam)5	tgtaat**GGATCC** ACTGCAGAGAGGCTTGAAAG
16	Pxks1(Kpn)3	cagtca**GGTACC** TGTGAGTGTCAACTACGTGAGT
17	Ptef1(Bam)5	tgactg**GGATCC** CCGTACACTTATTGTTAACTATGAA
18	Ptef1(Kpn)3	tgccat**GGTACC** TGTAGATAGACTTAGATTGTATGAAA
19	Padh1(Bam)5	actact **GGATCC** GAGGGAAAAACC
20	Padh1(Kpn)3	atgact**GGTACC**tGATAATTTGGATGGATCGCA
21	Pcyc1(Bam)5	tgactg**GGATCC** ATGGGATTTGTAGAAACTC
22	Pcyc1(Kpn)3	tgccat**GGTACC** TTTTGTGTGTAGTGAAGTTAATTG
23	Pccw12(Bgl)5	actact**AGATCT** CACAAAGAACCAACAAACTATG
24	Pccw12(Kpn)3	catact**GGTACC** CTAGTTTGAGTTAAGAAGTAGAGTG
25	Ppgk1(Bam)5	actact**GGATCC** GTGGAATCTGGTGATTGTCG
26	Ppgk1(Kpn)3	ctaact**GGTACC** TATGTAGAGATAGCGATGTGAATG
27	Phhf1(Bam)5	cataca**GGATCC** AGCGTACACAGCCGTTAGTC
28	Phhf1(Kpn)3	actact**GGTACC** TGTTACTGATTAGATATATGAGTTTG
29	EGC3us(SacI)5	actaca**GAGCTC** AGCTCAATCCGTACCACATG
30	EGC3(SacI)3	actaca**GAGCTC** TTTCAATATCTGATTCAATTCC
31	GSC2int(SacI)5	actaca**GAGCTC** GTTGACATACTTGACAGATTTGG
32	GSC2int(SacI)3	actaca**GAGCTC** AACTCCTGAACAGCCAAAG
33	CRF1us(SacI)5	actaca**GAGCTC** ATTGGGTGCCATAATCACAC
34	CRF1(SacI)3	actaca**GAGCTC** ACATCGGCAAATTGACCTC
35	YEcherry(Kpn)5	actact**GGTACC** ATGGTTTCAAAAGGTGA
36	YEcherry(Afl)3	actact**CTTAAG** TTATTTATATAATTCATCCATAC
37	Ptdh3(Age)5	tcatta**ACCGGT** CTCGTTAGTATTTCCGTGAAG
38	CBGint(Xho)3	actatc**CTCGAG** CCCCGGGGACTAAGTAGAACTTT CAAAGAGCCGCTCTTAAACTC
39	coCBGint(Sal)5	actacct**GTCGAC** AAAGTTCTACTTAGTCCCCGGGG TTGCAGCCATTTTGTGTAGC
40	Tadh2(Xba)3	tgccat**TCTAGA** TTCTGCCTTCTGAACGTTTG
41	CaCas9(Kpn)5	actaca**GGTACC** ATGGATAAAAAGTATAGTATTGG
42	CaCas9(Afl)3	actact**CTTAAG** TTATCACTTGTCATCGTCATC
43	Ptdh3(Sbf)5	tcatta**CCTGCAGG** CTCGTTAGTATTTCCGTGAAG
44	Ptdh3(Fse)3	atgact**GGCCGGCC** GATGAATTGTTTATAGGGAAGA
45	Tadh1(Mlu)5	cattcg**ACGCGT** GCTAGATAGTGCTTTGTTC
46	Tadh1(SacII)3	actact**CCGCGG** AGAGTGCATTGGTTAGGTGG
47	sgRNA5	AAACGGGCGTGTGGCG
48	sgRNA(Mlu)3	actact**ACGCGT** GTCCCATTCGCCATGC
49	YUM1(sgRNA)sap5	CCAAAAGTTCTACTTAGTCCCCG
50	YUM1(sgRNA)sap3	AACCGGGGACTAAGTAGAACTTT
51	Ptef(Bam)5	actact**GGATCC** GTACACTTATTGTTAACTATG
52	Tadh2(Spe)3	gttcac**ACTAGT** TCTGCCTTCTGAACGTTT
53	LacZint(Xba)5	actact**TCTAGA** CCATGATTACGGATTCACTGG
54	LacZint(YumBam)3	actata**GGAT** **CC**CCGGGGACTAAGTAGAACTTT ATCCGCCACATATCCTGATCT
55	LacZint(YumSpe)5	attacct**ACTAGT** AAAGTTCTACTTAGTCCCCGGGG ACCATGATTACGGATTCACTGG
56	LacZint(Afl)3	actact**CTTAAG** ATCCGCCACATATCCTGATCT
57	YEcherry (Fse)5	actact**GGCCGGCC** ATGGTTTCAAAAGGTGA
58	YEcherry (Mlu)3	actact**ACGCGT** TATTTATATAATTCATCCATAC
59	Pccw12(Sbf)5	tgacgt**CCTGCAGG** CACAAAGAACCAACAAACTATG
60	Pccw12(Fse)3	gtccat**GGCCGGCC** CTAGTTTGAGTTAAGAAGTAGAGTG
61	UraUSc(XhoEcoV)5	actaca**CTCGAGGATATC** AAAGAAAGAAGAAGCCATTACTG
62	UraUSc(Xba)3	actact**TCTAGA** TGTCGAATGTTTTTAGGGTG
63	UraDS(Nde)5	actact**CATATG** AAGAAGACAAGCCAAGTG
64	UraDSc(Sac2EcoV)3	actact**CCGCGGGATATC** GACATAAACAAGAAGTTTTCCG
65	URAusScrn5	GATGAGATTTCCTCCTTAGG
66	URAdsScrn3	CGATAGGAATGCTTGTCAATG
67	PsCBG(Kpn)5	TTCATC**GGTACC** ATGGTGAAGCGTG
68	PsCBG(NheEcoAfl)3	TATGCC**CTTAAG**AATTCGATCTA**GCTAGC** ACCGCCGGCCTTCTCCAAC
69	EGC3intA3	GATCTTCGTACCTGCAGTG
70	CRF1intB3	GTTACTTCCGTTCGAGTTTAC
71	URAusScrnC5	TGACGATAACTTACGAGAGG
72	PsKanScrn3	CGTGAGTCTTTTCCTTACCC
73	PsCBGscrn5	AAACAATTGTTGGAGAAGGC
74	URAdsScrnC3	GTTTGAGATCATGGATGGTG
75	URAseq5	GTTGCATCTCAAACAGTG
76	URAseq3	ATTGACTGACGACTTACTGG

^*a*^ Underlined portions of primers bind to the target. Bold portions are restriction sites. Lowercase indicates nonspecific nucleotides for restriction site spacing. Green letters represent YUM1 sequence.

### YUM1 Cas9 target design

The 23 base YUM1 sequence AAAGTTCTACTTAGTCCCCGGGG (underlined is the PAM) was designed as a Cas9 targeting sequence based on the sequence’s absence in the *S. stipitis* and *S. cerevisiae* genomes. The 3′ portion of YUM1 sequence “CCCCGGGG” was reported to not exist in the genome of *S. cerevisiae* (19) and also provided a PAM for the intended Cas9 targeting. We used that sequence as a seed for the keeSeek algorithm (20) to find 23-mers that were nonexistent in the *S. cerevisiae* genome. The Yeast Unique Motif 1 (YUM1) was chosen as the top candidate based on it also having smaller strings of nucleotides that were also nonexistent or rare in yeast genomes, and it not causing hairpins when included in primers 38, 39, 49, 50, 54, and 55 (see Table 2).

### Transformations

Chemically competent *E. coli* (TOP10) were transformed using a standard chemical transformation procedure. *S. stipitis* was transformed using a modified lithium acetate/PEG procedure. This consisted of growing a diluted culture of *S. stipitis* in 10 mL of YPD at 30°C shaking (180 rpm) until it reached OD_600_ of 0.8–1.0, pelleting the cells at <3,000 rcf, and washing the cells with 1 mL 1× TE, 0.1 M lithium acetate. The cells were pelleted and resuspended in 100 µL of 1× TE, 0.1 M lithium acetate, and incubated at 30°C rolling for 1 h. Then, 5–10 µg of linearized or circular plasmid DNA (in 20 µL) was added, along with 15 µL of 10 mg/mL herring sperm DNA (Promega, Madison, WI), and 700 µL of 40% PEG_8000_, 1× TE, 0.1 M lithium acetate. For the case of *ura3* gene deletion, 120 µg of linearized plasmid DNA (in 20 µL) was used. This mixture of DNA, cells, and PEG/TE/LiOAc was rolled at 30°C for 30 min, and afterward, the mixture was heat shocked at 42°C for 5 min, centrifuged for 10 s at 10,000 rcf, and the supernatant was removed by vacuum aspirator. Cells were resuspended in 3 mL of YPD and incubated at 30°C shaking for 3 h then plated on YPD containing appropriate antibiotic and 100 µM beetle luciferin (Promega, Madison, WI) and grown for 2–3 days at 30°C.

### PCR and PCR screening

Phusion high-fidelity DNA polymerase (Thermo Fisher, Waltham, MA) was used for PCR when CDSs were amplified for cloning purposes. For all other PCR, GoTaq (Promega, Madison, WI) was used. PCR was used to confirm *S. stipitis* genomic modification by BLINCAR. Introduction of pWR101, pWR102, and pWR103 (see Table 1) was confirmed using primer 57 paired with primers 69, 32, and 70, respectively (see Table 2). Targeting BLINCAR to replace the *URA3* gene was confirmed upstream by pairing primer 71 (which targets a genomic sequence upstream of the introduced element) with primer 72 (which targets a portion of the coKanMX CDS within BLINCAR). BLINCAR replacement of *URA3* was confirmed downstream by pairing primer 73 (which targets a portion of coCBG within BLINCAR) with primer 74 (which targets a genomic sequence downstream of the introduced element). Positive control PCR to confirm the presence of *S. stipitis* genomic DNA used either primers 75 paired with 76 to give a 1 kb product or primers 33 paired with 34 to give a 2 kb product.

### Imaging

Bioluminescent and brightfield imaging of yeast grown on solid media in 10 cm Petri dishes was performed using a ChemiDoc MP (Bio-Rad, Hercules, CA). Bioluminescent imaging was recorded using 4 × 4 binning for exposure times of 1–5 min. Microscopic imaging was performed using an Olympus BX60 fluorescent microscope and images were captured from a DP72 color CCD camera (Olympus, Tokyo, Japan). Fluorescence was achieved from a U-MWB filter cube (450–480 nm band-pass excitation filter, 500 nm dichroic mirror, and 515 nm long-pass emission barrier filter).

## RESULTS AND DISCUSSION

### Construction of a versatile bioluminescence reporter plasmid for *S. stipitis*

**Fig 1:**
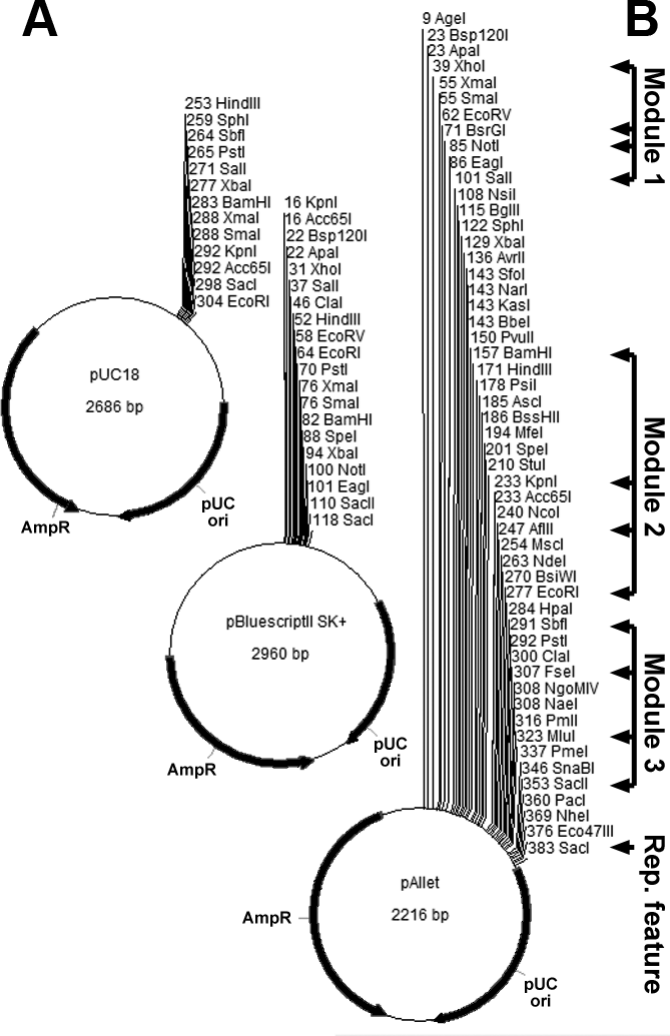
pAllet is a compact *E. coli* cloning vector with an extensive MCS. (**A**) pAllet’s size and MCS compared to two representatives from commonly used *E. coli* cloning vector series, pUC, and Bluescript. (**B**) Modules where three functional *S. stipitis* heterologous genes were constructed in pAllet. Arrows indicate restriction sites in the pAllet map where the promoter, CDS, and terminator (respectively) were introduced to assemble the heterologous genes for many of the plasmids constructed in this study. The single arrow at the bottom shows the restriction site where the replication/stabilization feature (e.g., ARS or integration locus) was added for many of the plasmids used in this work.

We desired a plasmid with room to sequentially clone 10 or more genetic elements [three coding sequences each with its own *S. stipitis* promoter and terminator, plus a plasmid-maintenance feature like an autonomous replicating sequence (ARS) or integrating element]. Since long, heterologous elements often contain problematic restriction sites that limit cloning designs, we realized the multiple cloning sites of common cloning vectors like pUC18 and pBluescript were too limited to provide the extent and flexibility we sought for our cloning platform, so we synthesized pAllet, a cloning vector with an extensive multiple cloning site (MCS) containing over 50 unique restriction sites (Fig. 1A). In our design of pAllet’s MCS, we sought to maintain the order of some of the common restriction sites found in the pUC and pBluescript series of plasmids so that elements which have been previously cloned by others into these common vectors could more seamlessly be transferred to pAllet if needed. Namely, the order of SalI, XbaI, BamHI, KpnI, and SacI was preserved from pUC18, while the order of ApaI, XhoI, SalI, HindIII, EcoRI, PstI, SacII, and SacI was preserved from pBluescriptII SK+. Another problem with common cloning vectors is that often useful restriction sites are immediately adjacent to one another or even overlapping in the MCS thereby not allowing efficient use of both sites simultaneously. To alleviate this problem in pAllet and extend the range of useful cloning sites, we interspersed less common sites and blunt-cutting sites between common staggered-cutting sites that are often used for cloning.

Through the course of this work, we used pAllet to construct over 100 derivative plasmids, drawing on a modular approach. Figure 1B shows modules 1, 2, and 3, where we introduced up to three heterologous genes. Adjacent arrows in each module point to restriction sites in pAllet (from Fig. 1A), where a gene element (promoter, CDS, or terminator) was introduced. SacI (bottom arrow in Fig. 1B) was used to provide the plasmid with a replication or integration element for stability in *S. stipitis*. We chose the restriction sites BsrGI, KpnI, and FseI as promoter-CDS junction sites for modules 1–3, respectively. These sites were chosen because their 6 bp recognition sequences end with three nucleotides that are commonly found immediately before the start codon of *S. stipitis* CDSs. Given that the nucleotide sequence near the start codon influences efficient translation in yeast (21), we surveyed sequences of 60 *S*. *stipitis* genes suspected to be regularly expressed in common growth environments to help inform our selection of these junction sites (see Table S2).

**Fig 2:**
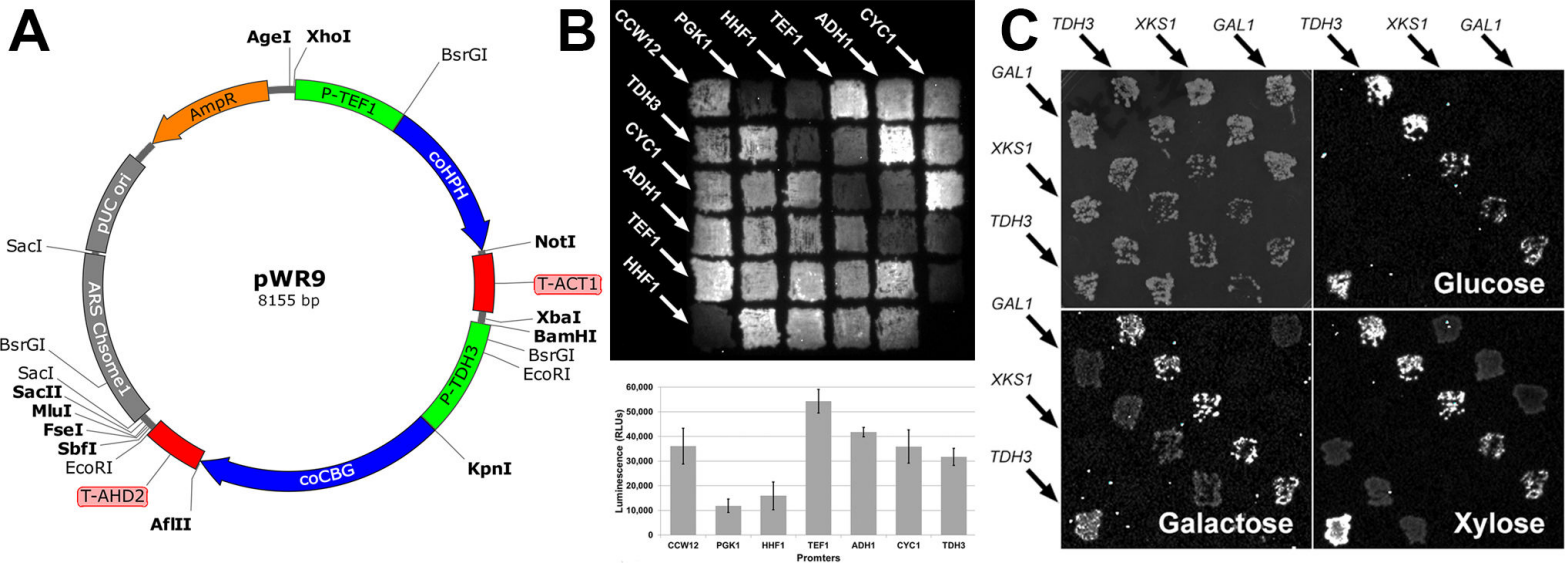
A hygromycin-selectable bioluminescent reporter was used to test intact heterologous gene activity and to reveal the strength of several constitutive and inducible promoters in *S. stipitis*. (A) Plasmid map of pWR9, the foundational bioluminescent shuttle vector used (and modified) for this study. Bold restriction sites are unique. The *TDH3* promoter driving coCBG expression was exchanged for other promoters shown in (B) and (C) using BamHI and KpnI. (B) Relative strengths of seven suspected constitutive *S. stipitis* promoters driving coCBG. Bioluminescence from a 10 cm Petri plate (top panel) shows patches from separate colonies of *S. stipits* transformed with derivatives of pWR9 with different promoters driving coCBG in diagonal rows indicated by the promoter’s gene name and arrows (3 min exposure). The bottom right of the 6 × 6 patched grid does not have yeast at that location and was used for background subtraction of pixel intensity quantification from each of the other patches (bottom panel). Bars show average relative luminescence (±S.D.) for each of the seven promoters tested (*n* = 5 patches). (C) The sugar-inducible promoters from *XKS1* and *GAL1* genes produce sugar-specific control of bioluminescence. *S. stipitis* transformed with pWR9 or derivatives where P*_TDH3_* P*_XKS1_* or P*_GAL1_* drive coCBG expression were grown as patches in diagonal rows on a 10 cm Petri plate (top left panel, brightfield) and replica-plated onto nutritive agar media containing 2% glucose, 2% galactose, or 2% xylose. Bioluminescent imaging for 5 min exposure (top right and lower panels) reveals that P*_TDH3_* promotes transcription of the coCBG reporter on all three sugars, but P*_XKS1_* only does so on xylose, and P*_GAL1_* only does so on galactose.

We constructed a basic versatile bioluminescent reporter episomal shuttle vector, pWR9 (see Fig. 2A), for *S. stipitis* by introducing native regulatory elements into pAllet to control the expression of the HPH hygromycin resistance gene in module 1 and the green bioluminescent reporter gene CBG99 in module 2, both of which had been codon optimized for *S. stipitis* to bypass the CUG codon restriction. We also added a 1.1 kb ARS found on *S. stipitis* Chromosome 1 (first reported as ARS2 by Yang et al. in 1994) for purposes of replication and maintenance as an episomal plasmid in *S. stipitis* (7). The unmodified CBG99 sequence from Promega’s pCBG99-basic plasmid (in which *S. stipitis* translates the CUG codons as serine instead of leucine) was insufficient to produce bioluminescence in *S. stipitis* (see Fig. S1). Therefore, codon-optimization for 18 CUG codons to UUG codons was necessary for bioluminescence in *S. stipitis*. Once we confirmed transformation, selection, and bioluminescence of pWR9 (see Fig. S1) we made derivatives of the basic reporter plasmid by swapping various elements to compare antibiotic selectable markers, promoter strength, replication elements, and plasmid stability (see Table 1).

In addition to pWR9, which used coHPH to confer hygromycin resistance, we also created pWR10, pWR23, and pWR24 to compare plasmid selection using codon-optimized versions of ShBle (resistance to zeocin), KanMX (resistance to G-418), and Nat (resistance to nourseothricin). Nourseothricin was found to be quite toxic to *S. stipitis* and coNat was unable to provide any resistance/selection regardless of the concentration of antibiotic. And although coShBle and coKanMX provided sufficient selection for early identification of transformed candidates in the first day or two of growth, an inherent natural resistance/tolerance to zeocin and G-418 allowed some false positive colonies to form and eventually overtake the plate (data not shown). Hygromycin was found to be sufficiently toxic to *S. stipitis* with little to no natural tolerance, and the resistance provided by coHPH produced a clear selection differential in transformants (no false positives) which persisted for days to weeks of growth after inoculation.

We used the coCBG gene in module 2 of pWR9 derivatives as a reporter gene to evaluate the relative strength of seven *S*. *stipitis* promoters whose homologs in *S. cerevisiae* have been shown to have a strong constitutive expression (22). Figure 2B shows bioluminescence and quantification from patches of colonies transformed with pWR9, pWR54, pWR55, pWR56, pWR77, pWR78, and pWR81, which used the promoters of *TDH3*, *CCW12*, *PGK1*, *HHF1*, *TEF1*, *ADH1*, and *CYC1* (respectively) to drive coCBG expression. The *TEF1* promoter produced the strongest bioluminescence. Promoters from *TDH3*, *CCW12*, *ADH1*, and *CYC1* all produced about the same moderate amount of bioluminescence, and promoters from *PGK1* and *HHF1* produced the least bioluminescence.

We also tested bioluminescence from *S. stipitis* transformed with pWR11 and pWR22, which were pWR9 derivatives that used the sugar-inducible promoters of *GAL1* and *XKS1* (respectively) to drive coCBG expression. As shown in Fig. 2C, bioluminescence (at a moderate level) was only produced from the *GAL1* promoter reporter when cells were grown on media containing galactose; and similarly, bioluminescence was only produced from the *XKS1* promoter reporter when cells were grown on media containing xylose. Taken together, these data show that pWR9 and its derivatives are useful reporters for evaluating promoter strength and conditional gene regulation of *S. stipitis* grown on solid media.

The data from Fig. 2 show that the ARS sequence from Chromosome 1 is sufficient to maintain the plasmid in replicating cultures under selection pressure; however, to test whether the plasmid can be maintained in the absence of antibiotic selection and whether genomic integration of the plasmid could allow maintenance of transgenic elements without selection, an experiment was conducted with episomal and integrated plasmids. pWR16 is a derivative of pWR9 in which we swapped the ARS element for a copy of the 2.7 kb *S*. *stipitis* genomic region containing the *URA3* gene (which also included a unique EcoRV site in the middle of this region that was used for plasmid linearization). Survival and bioluminescence from *S. stipitis* harboring the pWR9 episomal plasmid was compared to that of *S. stipitis* harboring the pWR16 integrated plasmid after growing overnight in YPD broth with and without hygromycin antibiotic. Figure S2 shows that the pWR9 episomal plasmid maintained by a chromosomal ARS was rapidly lost (within 12 generations) during culture if not kept under selective pressure; however, the pWR16 integrated plasmid was stably maintained in absence of selection. A linearized plasmid containing homology to an *S. stipitis* genomic sequence is capable of stably integrating into the host’s chromosome by way of HR. The difference in plasmid stability between these two maintenance elements is a phenomenon that we exploit for Cas9-based strain alteration later in this work.

### Bioluminescence to report Cas9 activity

A functioning Cas9 system requires properly produced Cas9 protein and small guide RNAs to be at sufficient concentrations to act on an intended DNA target. If any step in the production, assembly, or targeting phases of this system is insufficient, then Cas9 efficiency suffers. Two common approaches to demonstrate successful Cas9 activity are to use it to (i) nullify a detectable gene product by cutting a target gene that is briefly retracted before repair by NHEG, or (ii) repair a nonfunctioning gene to produce a detectable gene product by cutting a target gene that is then repaired by HR using a provided repair template. Both approaches have obstacles in organisms that are difficult to transform (like *S. stipitis*). Detecting a rare loss of function (especially if subtle or heterogeneous in a colony) can require screening hundreds of colonies, and often requires sequencing to confirm the loss of function was due to Cas9-targeted cuts and not spontaneous mutations. And repairing a nonfunctional reporter by repair template requires a successful co-transformation of the repair template along with the delivery of the other Cas9-dependent genetic elements.

**Fig 3:**
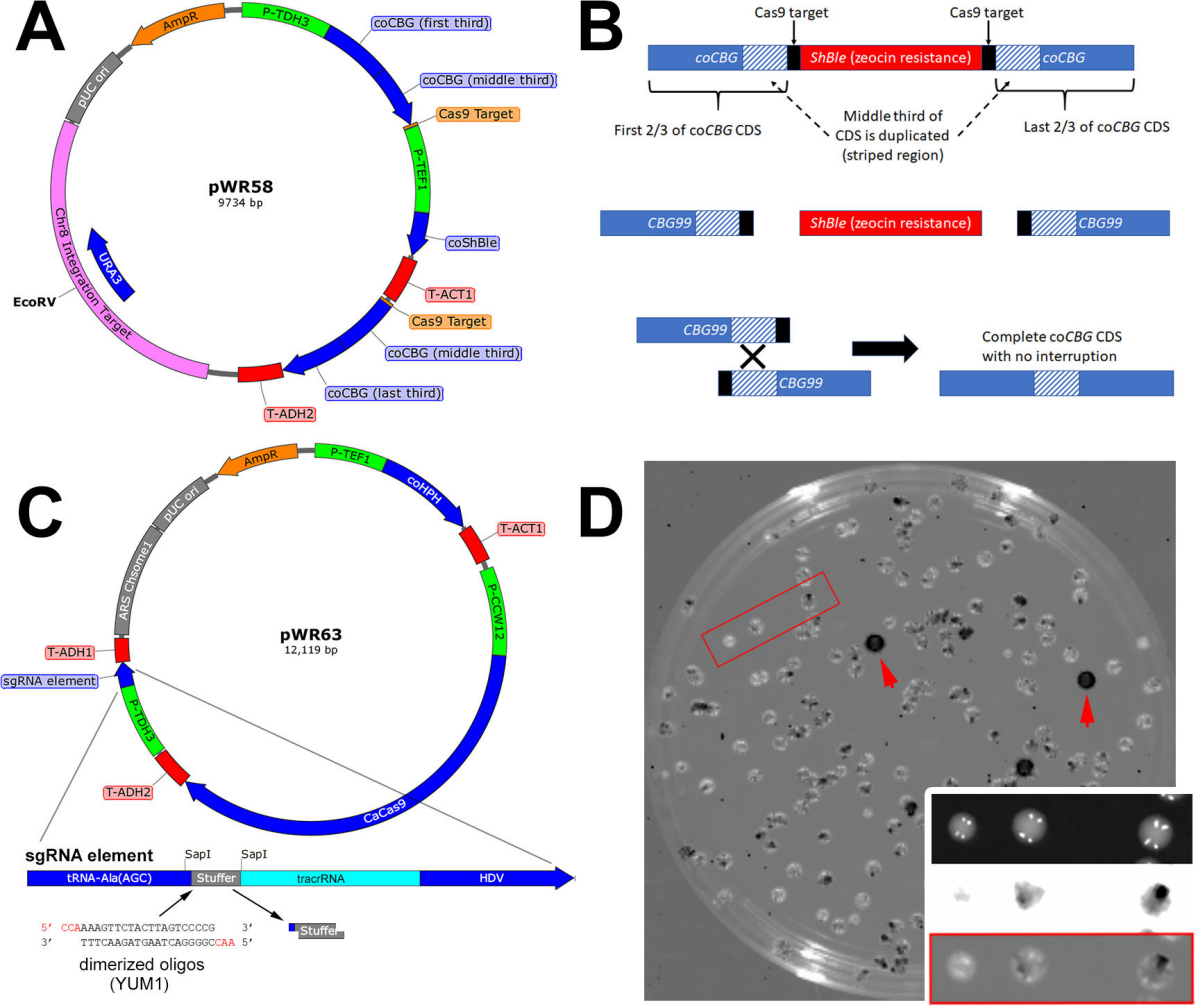
sgRNA-targeted Cas9 activity was confirmed in *S. stipitis* using pWR58, a positive bioluminescent reporter of Cas9-actuated recombination. (**A**) Map of pWR58, the bioluminescent Cas9 activity reporter which is linearized at the EcoRV site and integrated into *S. stipitis’s* genome. (**B**) Illustrates how pWR58, the bioluminescent Cas9 reporter, works. The top portion shows the integrated reporter before Cas9 activity. The coCBG element is interrupted by a selectable ShBle gene and therefore does not produce a bioluminescent product. The middle portion shows the resulting products after targeted-Cas9 has cut the reporter. The bottom portion shows homologous recombination between the repeated elements of coCBG resulting in an intact (non-interrupted) coCBG CDS which produces a bioluminescent protein. (**C**) Map of pWR63. The top shows the untargeted Cas9 and sgRNA expression vector. The bottom shows a closer view of the sgRNA element of pWR63 before being programmed with the user-designed sgRNA element. The dimerized oligos that form the YUM1 targeting sgRNA element replaced the “stuffer” region between the SapI restriction sites to make pWR63(YUM1), the functional plasmid that was used to affect the pWR58 reporter in *S. stipitis*. (**D**) The bioluminescent and brightfield overlay images of a 10 cm hygromycin selective plate of an *S. stipitis* pWR58 reporter strain transformed with pWR63(YUM1), i.e., the Cas9 and sgRNA expression plasmid. Bioluminescence (5 min inverted exposure) appears as black signal on a white background for purposes of visualizing it on top of its brightfield counterpart. Most colonies that received pWR63(YUM1) had a sectoring pattern, where Cas9 only affected a portion of the cells in the colony. The inset shows a closer view of this for three colonies highlighted by the red box. The top of the inset shows brightfield, the middle shows inverted bioluminescence, and the bottom shows the merge. However, some colonies were completely affected by Cas9 such as those indicated by red arrows.

To get around these obstacles, we augmented our bioluminescent reporter to create an integrating zeocin-selectable positive reporting system for Cas9 activity that requires only single selectable/detectable transformation events. The bioluminescence-based Cas9 reporter, pWR58, can be stably integrated into the *S. stipitis* genome and selected by growth on zeocin-containing media. In its initial state, pWR58 does not produce a bioluminescent product because its luciferase gene (coCBG) contains an interruption cassette (i.e., the zeocin selectable marker) nested within a coCBG internal duplication where the middle third of the coCBG CDS is duplicated on both sides of the interruption cassette (see Fig. 3A). If functional Cas9 is present and contains the necessary sgRNA sufficient to target a designed artificial 20 bp DNA target (yeast unique motif, “YUM1”) which is natively absent in the *S. stipitis* genome that flanks the interruption cassette, then Cas9 can cut the integrated reporter at either (or both) of these target sequences, thereby spurring HR to repair the break by recombining the coCBG within its duplicated middle third and forming an intact, complete coCBG CDS (see Fig. 3B).

To provide *S. stipitis*, the necessary components to affect the integrated pWR58 reporter plasmid (namely a CUG-yeast-optimized Cas9 gene and a sgRNA encoding sequence), we created pWR63 (see Fig. 3C). This versatile episomal plasmid contained the CaCas9 CDS (18) optimized for *C. albicans* (which bypasses the CUG codon restriction in CUG group yeast like *S. stipitis*) driven by the *CWW12* promoter from *S. stipitis* determined in this work to be moderately strong and constitutive (see Fig. 2B). Guide RNA expression for many engineered Cas9 systems is driven from POL III-type promoters like the *SNR52* promoter, but confirming the boundaries and strengths of these types of promoters can be challenging and labor-intensive since their RNA products are not translated. To make a versatile sgRNA expression system that uses RNA expressed from *S. stipitis* POL II-type promoters (like those tested and confirmed in Fig. 2), we adopted a sgRNA production system published by Ng and Dean, where the transcribed targeting crRNA/tracrRNA element is preceded by a tRNA sequence and followed by a hepatitis delta virus (HDV) ribozyme sequence (23). Once transcribed by POL II, the tRNA portion of the RNA is cleaved and removed by native RNAse Z (24) and the HDV portion is cleaved and removed by self-cleavage (25), leaving a functional sgRNA with precise (and uncapped) 5′- and 3′-ends. Dimerized oligos bearing the desired 20 nucleotide sgRNA-encoding sequence and three base overhangs can be added to pWR63 by cutting the plasmid with SapI, removing a small “stuffer” sequence and producing corresponding three-base overhangs for directional cloning of the dimerized oligo (see Fig. 3C). For this work, we introduced the sgRNA encoding sequence necessary to target the artificial YUM1 target sequence. We called this derivative pWR63(YUM1).

The pWR58 reporter, once affected by Cas9 [from pWR63(YUM1)], produced a bioluminescent product that could be visually detected in colonies by a chemiluminescence imaging camera (see Fig. 3D). A strain of *S. stipitis* which had been stably transformed with the pWR58 reporter plasmid was subsequently transformed with the pWR63(YUM1) episomal plasmid to produce Cas9 and cut the YUM1 targeting elements in the pWR58 construct. After a 3-h recovery period post-transformation, these yeasts were plated on a YPD nutrient plate containing hygromycin [which selected for the pWR63(YUM1) plasmid] and luciferin (the substrate for the luciferase). Figure 3D shows that some of the transformed yeast grew into solid, intensely glowing colonies (e.g., Fig. 3D, red arrows), while other transformed colonies exhibited a sectoring phenotype or did not show detectable levels of bioluminescence at all (see Fig. 3D inset which shows the three colonies contained in the red box). Sectoring could result if colony-forming units were comprised of more than one cell in which only a subpopulation (or a single cell) of those cells underwent the Cas9-directed reporter gene modification. Alternatively, the Cas9-directed reporter gene modification could have occurred after plating in one cell of the early growing colony. Transformation with a plasmid that lacked either the YUM1 encoding sgRNA sequence or the CaCas9 gene resulted in colonies that survived selection but were not bioluminescent (see Fig. S3). This assay confirmed that Cas9 and the guide RNA were expressed in *S. stipitis* at sufficient levels to target and cut the artificial target sequence in the reporter and that HR repaired the broken chromosome in instances that were easily identifiable.

### BLINCAR for iterative additions to the wild-type *S. stipitis* genome

Given that wild-type *S. stipitis* is only susceptible to the toxicity of a few antibiotics (e.g., hygromycin, zeocin, and G-418) for which codon-optimized resistance genes have been found to circumvent, there are not many selectable markers available if one desires extensive augmentation to the wild-type *S. stipitis* genome. However, the Cas9 actuated recombination that was shown between the coCBG repeats flanking a selectable marker (Fig. 3) highlighted a way the limited set of selectable markers could be reused for potentially unlimited genomic modification.

**Fig 4:**
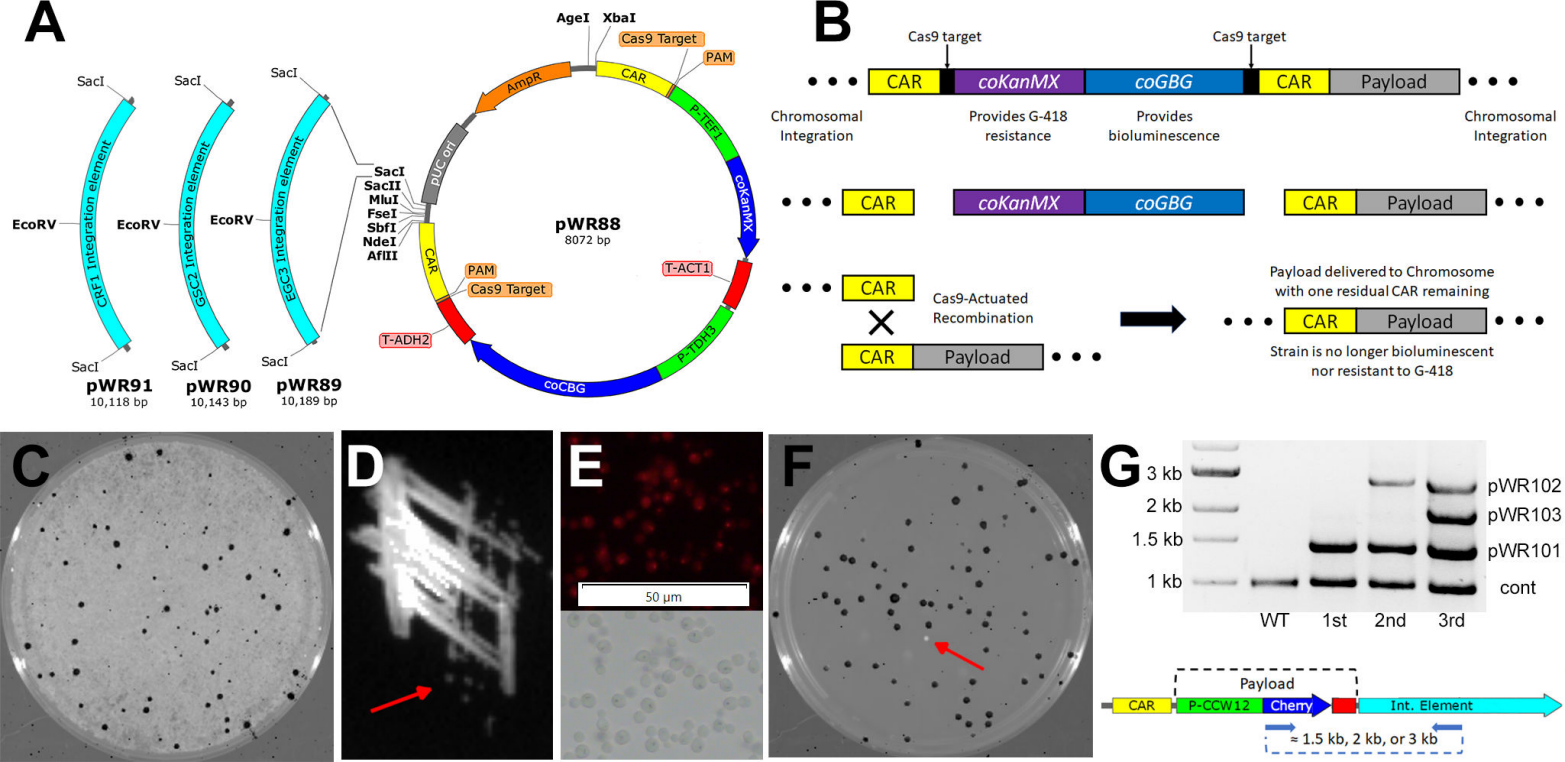
BLINCAR was used to integrate three separate copies of a mCherry expression cassette as an example of repeated payload delivery to the *S. stipitis* genome. (A) Map of pWR88, the core BLINCAR payload delivery plasmid. Three derivative plasmids of pWR88 involving different *S. stipitis* integrating elements added at the SacI site are also shown to the left of the map. The name and size of each derivative plasmid are shown under the corresponding integration element. The EcoRV site in the center of each integrating element is the site for linearizing the construct. (B) Illustrates how the selectable and bioluminescent elements of BLINCAR are removed once integrated leaving the integrated payload in the genome. Such a strain is susceptible to repeated use of a BLINCAR delivery plasmid. (C) The bioluminescent and brightfield overlay of a 10 cm G-418 selective plate of *S. stipitis* transformed with linearized pWR101 (a derivative of pWR89 that carries a mCherry expression cassette). Bioluminescence (5 min inverted exposure) appears as black signal. (D) Bioluminescence (1 min exposure) for single colony isolation where a portion of a 10 cm (nonselective) plate was streaked with one glowing colony from the plate in (C). The red arrow shows the single colony that was chosen for further work. (E) Red fluorescence (top) and brightfield (bottom) micrographs of *S. stipitis* transformed with pWR101. Images were captured at 40× objective magnification with 75 ms exposure. The 50 μm scale bar is shown in white. (F) shows the bioluminescent and brightfield overlay of a 10 cm hygromycin selective plate of single colony isolate from C transformed with pWR63(YUM1), the Cas9, and sgRNA expression plasmid, to remove the coCBG and coKanMX genes from the integrated element. Bioluminescence (5 min inverted exposure) appears as black signal on top of its brightfield counterpart. An instance for which Cas9-actuated recombination removed the coCBG gene appears as a white colony (i.e., no bioluminescence) indicated by the red arrow. This colony was chosen for further work. (G) PCR screen results (top) for a wild-type strain of *S. stipitis* and three rounds of sequential addition of the mCherry payload (diagramed bottom). Blue arrows in the diagram show primer binding sites to generate PCR products of varying sizes (approximately 1.5, 2, or 3 kb) depending on the integration element used for that round. A control set of primers (cont) was used to amplify a 1 kb portion of the *URA3* gene from all candidate strains. The first round of treatment (1st) delivered pWR101, detectable by a 1.5 kb band. The second round (2nd) delivered pWR102, detectable by a 3 kb band. And the third round (3rd) delivered pWR103, detectable by a 2 kb band.

To demonstrate this potential, we developed pWR88, a versatile, reusable plasmid for integrating desired genetic payloads repeatedly to the *S. stipitis* genome (Fig. 4A). This plasmid consisted of a selectable and detectable element, comprised of a codon-optimized G-418 resistance gene (coKanMX) and the coCBG luciferase gene, flanked by two 0.6 kb heterologous repeats intended for Cas9 actuated recombination (the 0.6 kb repeats were designated “CAR” sequences). We chose three native *S. stipitis* genes (*EGC3*, *GSC2*, and *CRF1*) in which to target the plasmid’s integration for this demonstration. These genes were chosen for integration targets because duplication of these genes (a consequence of this form of gene-targeted integration) was predicted to be well-tolerated and not cause adverse phenotypes. Moreover, they were chosen because they each possessed a single EcoRV restriction site that could be used for plasmid linearization. Sections of DNA of approximately 2 kb containing each of these genes (*EGC3*, *GSC2*, and *CRF1*) were PCR amplified from wild-type *S. stipitis* and individually cloned into pWR88 at its SacI site to create the integrating derivatives pWR89, pWR90, and pWR91, respectively (Fig. 4A). Genetic payloads can be added to these derivatives on either side of the CAR regions using one or more of the unique restriction sites remaining in the plasmid (indicated in bold in Fig. 4A).

Figure 4B illustrates how pWR88 derivatives can be used repeatedly to add multiple genetic payloads to the *S. stipitis* genome. Once linearized and transformed, the entire plasmid (including payload) integrates into the *S. stipitis* chromosome through HR. And although rare, successful integration produces cells that exhibit G-418 resistance and bioluminescence (Fig. 4B, top). If a second, separate genetic payload needs to be added to the modified strain, the antibiotic resistance and bioluminescence markers from the first integration can be removed by Cas9 cutting YUM1 targets near the CAR regions of the integrated plasmid (Fig. 4B, middle). Once cut, HR between the CAR regions repairs the broken chromosome, excluding the G-418 selectable marker and bioluminescence gene while leaving in place one CAR element and the genetic payload (Fig. 4B, bottom).

We demonstrated repeated transgene delivery using a genetic payload of the mCherry gene codon-optimized for *C. albicans* (17) under the control of the *S. stipitis CWW12* promoter. The coCherry gene was cloned into pWR89, pWR90, and pWR91 plasmids between their SbfI and SacII sites to create the derivatives pWR101, pWR102, and pWR103, respectively. Successful transformants of pWR101 were selected on YPD plates containing G-418 and luciferin; however, after 2 days of growth at 30°C, a background of non-transformed cells grew up around the transformed colonies (Fig. 4C). Bioluminescence from the transformants, however, allowed us to discriminate positive colonies and streak for single transformed colonies. This was done in case the initial sampling of the colony was inadvertently mixed with non-transformed cells (Fig. 4D). The single colony identified by the red arrow in Fig. 4D was subcultured for confirmation and continued work. The presence of the coCherry gene was confirmed in the strain’s genomic DNA by PCR (data not shown) and evidence of red fluorescence was detected from the cells (Fig. 4E; Fig. S4). After confirmation of coCherry delivery, the selectable and bioluminescence markers were removed from the genome by transforming the culture with pWR63(YUM1) and selecting for transformants on YPD plates containing hygromycin and luciferin. Successful removal of the G-418 resistance marker and coCBG bioluminescence gene by Cas9-actuated recombination appeared as non-glowing colonies on the hygromycin selective plates (Fig. 4F, red arrow). The process was repeated for the introduction of pWR102 and pWR103 subsequently. Retention of each iteration’s addition of coCherry was confirmed by PCR using a 5′ primer specific for the coCherry transgene and a 3′ primer specific to each iteration’s integration element (either EGC3, GSC2, or CRF1) such that each iteration would produce a PCR product of a different size (Fig. 4G). The first iteration contained only coCherry delivered by pWR101. The second iteration contained that from pWR101 but also the one provided by pWR102. And the third iteration contained coCherry from all three (pWR101, pWR102, and pWR103). Red fluorescence from these modified strains appeared to increase in intensity with the addition of one and two copies of coCherry, but the addition of a third copy did not seem to increase fluorescence above that provided by two copies (see Fig. S4).

### BLINCAR for marker-less gene deletions in wild-type *S. stipitis* genome

Strain engineering goals often benefit from the removal of multiple genes from the microbe’s genome, but established CRISPR Cas9 methods for repeated gene disruption are problematic in wild-type strains that (i) have only a few selectable markers, (ii) resist transformation, and (iii) predominantly repair double-stranded DNA breaks by rapid/efficient NHEJ. We tested whether our BLINCAR approach could permit targeted gene disruption in conjunction with rapid identification of genomic modification followed by marker removal. For proof-of-concept, we targeted *S. stipitis’s URA3* gene for removal.

**Fig 5:**
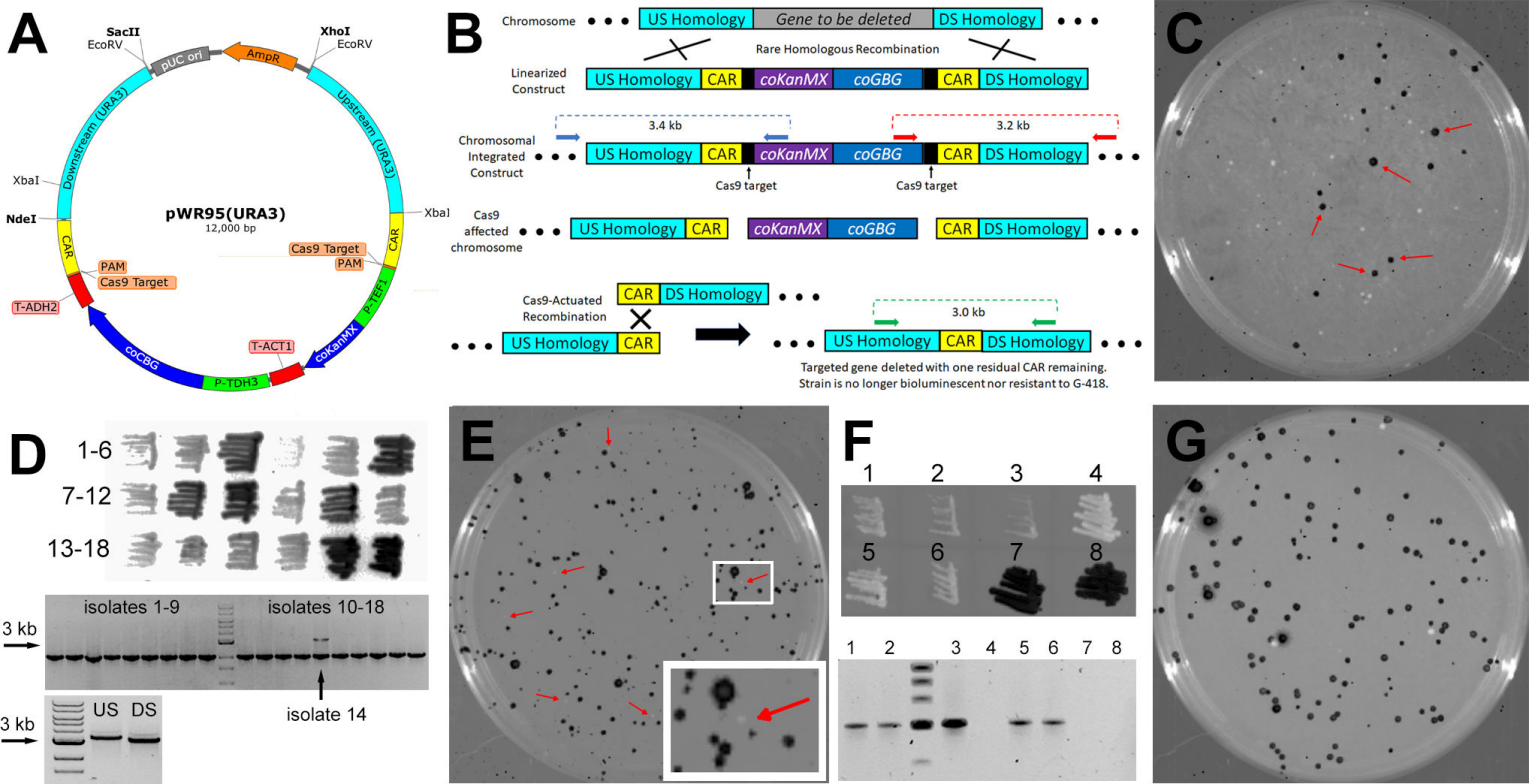
BLINCAR was used for targeted gene deletion of *URA3* from wild-type *S. stipitis*. (A) Map of pWR95(URA), the pWR95 gene deletion BLINCAR plasmid with 2 kb of homology upstream and downstream of the genomic *URA3* CDS added to the plasmid with XhoI, XbaI, NdeI, and SacII as shown. EcoRV linearized the *URA3*-targeting BLINCAR construct at the edges of *URA3* homology. (B) Illustrates how the BLINCAR construct works. Blue, red, and green arrows show primer binding locations for PCR used to confirm genomic modifications. Colored dotted lines show PCR product size for similarly colored primer sets. (C) The bioluminescent and brightfield overlay of a 10 cm G-418 selective plate of *S. stipitis* transformed with linearized pWR95(URA). Bioluminescence (5 min inverted exposure) appears as black signal. Colonies showing solid bioluminescence (examples shown by red arrows) were patched as isolates on a separate G-418 selective plate. (D) Inverted bioluminescence (1 min exposure) of 18 *URA3*-deleted candidate colonies (from C) patched as isolates in the upper panel. Numbers indicate isolate identity from left to right on the plate for that row. The middle panel shows results from a preliminary genomic PCR screen of the 18 isolates using two primer sets. One primer set targeted a 2 kb region of unaffected genomic sequence as a positive DNA control (for which all isolates showed a band). The other set (indicated by blue arrows in B) targeted a 3.4 kb element that would only arise if the BLINCAR construct was properly integrated into the *URA3* locus. Isolate #14 showed this 3.4 kb band, so integration at upstream (US) and downstream (DS) ends was confirmed by PCR (lower panel) using primer sets (blue and red arrows in B, respectively). (E) The bioluminescent and brightfield overlay of a 10 cm hygromycin selective plate of isolate #14 (BLINCAR::Δ*ura3*) transformed with pWR63(YUM1), i.e., the Cas9 and sgRNA expression plasmid, to remove the coCBG and coKanMX genes from the integrated element. Bioluminescence (5 min inverted exposure) appears as black signal on top of its brightfield counterpart. The inset shows a closer view of the section in the white box. Colonies for which Cas9-actuated recombination removed the coCBG gene appear as white colonies (i.e., no bioluminescence) indicated by red arrows. (F) The bioluminescent (1 min inverted exposure) and brightfield overlay of six non-bioluminescent colonies and two bioluminescent colonies (from E) isolated as patches on a separate hygromycin selective plate in the upper panel. Numbers indicate isolate identity. The lower panel shows the genomic PCR screen results of the eight isolates using the green primer set in B. The unnumbered lane is a size-standard where the darker band is 3 kb. (G) The bioluminescent (5 min inverted exposure) and brightfield overlay of a 10 cm selective plate lacking uracil of isolate #1 from F (CAR::Δ*ura3*) transformed with pWR96, a bioluminescent rescue plasmid bearing a functional copy of *URA3*.

To accomplish this, we designed pWR95, a modified version of pWR88 where sections of upstream and downstream genomic homology (that flank an intended target gene) can be directionally introduced on either side of the BLINCAR element. Approximately 2 kb of *URA3* upstream homology was added to pWR95 at the plasmid’s unique XhoI and XbaI sites, and a similar length of *URA3* downstream homology was added at the unique NdeI and SacII sites. EcoRV sites (which were otherwise absent) were added to the extremes of these homology portions for purposes of extracting the linear homology-flanked BLINCAR element from the plasmid for transformation into *S. stipitis*. Figure 5A shows pWR95(URA3), this pWR95 derivative with *URA3* homology flanking the BLINCAR element.

Some yeasts like *S. cerevisiae* are much easier to transform than *S. stipitis* (requiring only a few micrograms of DNA), and efficiently undergo homologous recombination allowing much shorter arms of homology be used for their gene integration cassettes (in some cases as short as 40 bp). For such yeast, it is common to produce gene integration cassettes using single reaction PCR, or even overlap extension PCR. However, in our hands, *S. stipitis* only reliably transformed with gene-targeting integration when we used large amounts of DNA (60–120 µg) and much longer arms of homology (1500–2000 bp). Transformation and targeting frequencies correlated with greater amounts of DNA and longer homology arms used. This performance was consistent with reports by others (2). Because our linearized BLINCAR gene targeting cassettes were about 10 kb long, and because we required 100 µg or more of DNA per transformation, we chose to use a plasmid-based approach rather than PCR to construct and produce the gene integration cassettes. This allowed us to produce large amounts of our long constructs in *E. coli* and purify them at high concentrations with maxiprep techniques.

Figure 5B shows how BLINCAR gene disruption is theorized to work. We linearized the construct and transformed it into wild-type *S. stipitis* where rare double HR events replaced the targeted gene (*URA3*) with the portion of the BLICNCAR construct between the regions of targeted homology (Fig. 5B, top). Successful transformants were G-418 resistant and bioluminescent (Fig. 5C). Eighteen candidate colonies were patched for clonal isolation (Fig. 5D, top) and were PCR-screened for targeted integration using primers that target portions of the chromosome outside of the homology paired with those that target regions within the BLINCAR element (Fig. 5B, middle). All candidates produced a positive control PCR product (2 kb) however only isolate #14 produced the 3.4 kb PCR product showing evidence of targeted integration (Fig. 5D). For isolate #14, PCR was used to confirm the integration of both upstream (3.4 kb product) and downstream (3.2 kb product) portions of the BLINCAR construct (Fig. 5D, bottom). Once gene replacement had been confirmed, the strain was transformed with the episomal plasmid pWR63(YUM1) which provided Cas9 and guide RNA that cut the BLINCAR construct once or twice (Fig. 5B, middle) and allowed HR between CAR regions to repair the chromosome (Fig. 5B, bottom). Candidate colonies that had likely undergone such Cas9-actuated recombination were identified by loss of bioluminescence (Fig. 5E, red arrows). Six candidate colonies were patched for isolation and confirmation of stable loss of bioluminescence (Fig. 5F, top, isolates 1–6) along with two colonies that retained their bioluminescence (Fig. 5F, top, isolates 7 and 8). To confirm the removal of the selectable marker and bioluminescence gene, genomic DNA from isolates was PCR-screened using a pair of primers that targeted central portions of the upstream and downstream *URA3* homology (Fig. 5B, bottom and Fig. 5F, bottom), and sequenced to show a remaining CAR site where *URA3* used to be.

The sensitivity of *S. stipitis* to G-418, hygromycin, and uracil auxotrophy was tested for each of the three phases of the BLINCAR *URA3* replacement process (see Fig. S5). Phase 1 was the wild-type strain after stable integration of pWR95(URA3). Phase 2 was the strain after transformation with the pWR63(YUM1) episomal plasmid. And phase 3 was the strain after the pWR63(YUM1) episomal plasmid was cured from the strain. A fivefold serial dilution for wild-type *S. stipitis* and that for each phase of treatment was plated on YPD where each grew normally. However, when the same array of yeast was plated on YPD containing antibiotics or minimal media lacking uracil; only yeast from phase 1 grew in the presence of G-418, only yeast from phase 2 grew in the presence of hygromycin, and only wild-type *S. stipitis* grew in the absence of uracil, even after 3 weeks of growth. When uracil was supplemented into the minimal media, all yeast grew; however, those with the *URA3* gene removed grew very slowly, requiring several weeks to attain cell density observable on the plate. These yeasts exhibit growth as expected for each stage of modification, in agreement with the model that (i) a G-418 antibiotic resistance marker replaced the *URA3* gene thereby conferring uracil auxotrophy, (ii) a Cas9 expression plasmid provided temporary hygromycin resistance while Cas9 targeted the G-418 resistance marker for removal, and (iii) the Cas9 expression plasmid was eliminated from the yeast resulting in a strain that was *ura3* deficient and re-sensitized to G-418 and hygromycin. Finally, we demonstrated that this *ura3* auxotrophic strain could be used for *URA3* selection for pWR96, a derivative of pWR9 which was a bioluminescence plasmid bearing a functional copy of *URA3* (Fig. 5G).

### Conclusions

Although some approaches using CRISPR/Cas9 technology allow seamless genome editing that does not require selection markers to promote successful gene modification [e.g., see reference (14)], such approaches in *S. stipitis* have the drawbacks of low efficacy in wild-type strains or require specialized/engineered host strains to improve efficiency. Seamless/markerless gene alteration typically requires DNA sequencing to identify and confirm successful alteration, and if the genetic modification occurs at low frequencies, then researchers must sequence many candidates just to find one successful alteration. Our approach was to use antibiotic resistance genes as selectable markers for genetic alterations rather than seamless gene editing. We acknowledge that genetic alterations through this method may be rare in wild-type strains of *S. stipitis*, but for those wanting to work with a wild-type strain, antibiotic resistance markers offer three advantages: (i) selection for candidates that possess (or have lost) the marker thereby minimizing the impact for low frequency events by allowing those candidates to either outperform their counterparts or display a detectable phenotype, (ii) retention/maintenance of the genetic trans-element allowing the user to control the environment under which interruption cassettes or plasmids are retained or lost in the host strain, and (iii) limited protection of the culture from contaminating microbes that are not resistant to the antibiotics used.

However, using antibiotics and antibiotic resistance markers also comes with drawbacks. First, antibiotics themselves can be costly, and maintaining plasmids in *S. stipitis* requires constant selection pressure therefore constant antibiotic use. Second, without proper care in the laboratory, using antibiotics in research can contribute to antibiotic resistance in environmental pathogenic microbes which negatively impacts society. Furthermore, making complex genetic alterations to one’s host strain can require the use of multiple antibiotics and can require the creation of a multi-drug-resistant microbe. Our work sought to gain the benefits of using antibiotic selectable markers to promote intentional alterations to wild-type strains of *S. stipitis* while minimizing the drawbacks by removing all selectable markers in the strain after the alterations were made and confirmed.

This work contributes to the growing pool of molecular tools the research community has for investigating and manipulating CUG group yeast like *S. stipitis* and *C. albicans*. And although our work focused specifically on *S. stipitis*, some of the products (like pAllet) are broadly applicable to molecular biology research in general, and many other products from this work can easily be made to function in other yeasts (like *S. cerevisiae*) by swapping out promoters or in some cases replication features. The modular approach we took in constructing the plasmids from this work makes these alterations possible using traditional restriction enzyme-based cloning methods.

In this work, we produced pAllet, a small, high-copy cloning vector with an MCS containing over 50 unique restriction sites. We used pAllet to build the diverse set of molecular tools in this work, but it can benefit many other researchers who need a cloning vector with an extensive MCS.

First, we produced pWR9, a versatile bioluminescence reporter for *S. stipitis* to evaluate three things. We evaluated the stability of episomal maintenance versus chromosomal integration and found that integration allows stable maintenance of the introduced plasmid without the need for selection; however, the episomal plasmid (maintained by ARS1) was rapidly lost if not kept under selective pressure. Second, we used pWR9 to test candidate antibiotic resistance genes in *S. stipitis* and found that the coHPH sequence provides strong and discriminative selection on hygromycin*,* while coKanMX provides moderate early selection that wanes after a few days of growth on plates. Third, we used pWR9 to evaluate candidate promoter strength and conditional expression, for which we go on to use those confirmed promoters to drive the expression of other heterologous genes in later plasmids.

We produced pWR58, an integrating, zeocin-selectable, positive bioluminescence reporter for Cas9 activity in *S. stipitis*. We used it to demonstrate the sufficient expression, activity, and cooperation of Cas9 and the Pol-II-derived sgRNA elements to target our artificial YUM1 sequence in *S. stipitis*. The pWR58 plasmid could be modified by others to test similar components and sgRNA targets of interest in *S. stipitis* (besides YUM1), as well as modified by others to function in other yeasts besides *S. stipitis*.

Finally, we produced BLINCAR, a two-component system where first one delivers an antibiotic selectable/bioluminescent element that can be used to either provide an exogenous genetic payload to the *S. stipitis* genome or can be used to replace a native (non-essential) genetic element thereby deleting the targeted sequence; then secondly, one introduces a temporary, episomal plasmid that produces Cas9 and sgRNAs to target the selectable/bioluminescent element for removal. Such a system can be used repeatedly to extensively modify the *S. stipitis* genome. We demonstrated this potential by repeatedly adding additional copies of coCherry to the genome, and later by deleting the *URA3* gene and re-sensitizing the deleted strain to all antibiotics used.

## SUPPLEMENTAL MATERIAL

Supplemental Material msphere.00224-23-s0001.pdf Supplemental figures, tables, methods, and sequences.

ASM does not own the copyrights to Supplemental Material that may be linked to, or accessed through, an article. The authors have granted ASM a non-exclusive, world-wide license to publish the Supplemental Material files. Please contact the corresponding author directly for reuse.
