# Supplementary material for: BLINCAR: a reusable bioluminescent and Cas9-based genetic toolset for repeatedly modifying wild-type Scheffersomyces stipitis
Source: mSphere. 2023 Jun 22;8(4):e00224-23. doi: 10.1128/msphere.00224-23 (PMC10449509; doi:10.1128/msphere.00224-23)
Supplement: Supplemental Material — Supplemental figures, tables, methods, and sequences. [file msphere.00224-23-s0001.pdf]

Supplemental Materials_Goffredi et al.

***Osedax*-associated Campylobacterales**

The recovered *Osedax*-associated Campylobacterales belonged to the family *Arcobacteraceae,* and the genera *Sulfurospirillum* and *Sulfurimonas*. We use the more inclusive family name *Arcobacteraceae* for the barcoding results, since the genus *Arcobacter* has been reclassified recently into at least 6 additional genera (50). There appeared to be at least 4 *Arcobacteraceae* species level clades; several related to ribotypes recovered from *Osedax* previously (11), one additionally related to the chemoorganotroph *A. nitrofigilis* (51), and one from the Davidson whalefall *Osedax* was uniquely related to those recovered from the lugworm *Arenicola* and a Breviatea protist (Figure 2: ref. 52). A single *Sulfurospirillum* ribotype was closely related to the chemoorganotroph *S. arcachonense* isolated from marine sediments and some found in association with whalefall bones and sediment (2, 19, 53). Two species level *Sulfurimonas* clades were present, according to barcode analysis, and most closely related to a sulfur-oxidizing chemolithoautotroph recovered from hydrothermal vent surfaces (*S. autotrophica*; 54), animal-associated species, including *S. paralvinellae* recovered from the 'nest' of a hydrothermal annelid (55), and ribotypes recovered from *Alvinoconcha* snails (56-57; Figure 2). Based on 16S rRNA gene amplicon sequencing, Campylobacterales could be recovered from the mucous tube of some individuals, but represented a much smaller fraction of the community than on the trunks directly (< 30%; n = 5).

Figure S1: Bacterial community composition, based on 16S rRNA sequences, associated with the trunk tissue of 3 *Osedax* species from northern California, USA. This data is a subset of 11 worms for which only trunk tissue was analyzed, however delineation between the trunk and the root system was not always clear, and thus the recovery of 16S rRNA genes of the primary endosymbiont (shown as gammaproteobacteria), which resides in the root tissue, sometimes occurred.

Figure S2: Fluorescence microscopy of additional *Osedax* species showing various structural integration with epibionts. (A) O. *talkovici* (from dive DR1105), showing results from the general Eub338 probe, attached to Alexa488, and counterstained with DAPI. (B) *Osedax*

packardorum (from dive DR1105), showing results from a DAPI illumination only. (C) *Osedax packardorum* (from dive DR1105), showing results from the epsilon specific EPS549, attached to the Cy3 probe, and counterstained with DAPI. Arrows indicate bacteria within epidermal cavities. All scale bars 20 µm.

Figure S3: Microscopy of *Osedax lonnyi* showing bacteria associated with the mucous tube. (A) Light microscopy of a 5-µm Wright-stained section of a specimen from dive H1825, embedded in Steedman's resin. (B) Fluorescent image of the same specimen, stained only with DAPI. Bacterial cells are denoted by the arrows, while the DAPI-stained nuclei of the worm epidermis are denoted by the arrowhead. Scale bars 100 µm. (C,D) same region with DAPI and epsilon specific EPS549_Cy3 probe, resp, showing no signal. Scale bars 10 µm.

Figure S4: Fluorescence microscopy of additional *Osedax* species showing various structural integration with epibionts. (A-B) *O. lonnyi* specimen from dive H1825 (20-month time frame), using probes EPS549_Cy3 and Eub338_Cy5. Complete overlap between the probes is shown in dark orange. (C-D) *O. frankpressi* specimen from dive T991 (at 51-month time frame), using probes EPS549_FITC and Eub338_Cy3. Complete overlap between the probes is shown in yellow, in addition to DAPI-stained host cell nuclei in blue, with slight autofluorescence in the green channel, useful to visualize the long epidermal folds, with pockets filled with bacteria (arrow) within the mucous covering. All scale bars, 10 µm.

Figure S5: Non-metric multidimensional scaling (NMDS) ordination of microbial communities associated with *Osedax*, according to (A) host species, (B) specific whalefall location, and (C) time frame. Each point represents all Campylobacterales 16S rRNA sequences recovered from a single specimen. Displayed data was square root transformed.

Figure S6: Relative abundance of the 16S rRNA gene for the genera *Arcobacter, Sufurospirillum* and *Sulfurimonas*, recovered from barcoding of 6 *Osedax* species at 2 whalefalls off of northern California, USA. (A) Before normalization (data shown in Figure 5). (B) After normalization for rRNA copy numbers among the 3 genera (ranging from 2-12), revealed by metagenomic sequencing (Table 2). A comparison of the statistical significance of each taxon-

time frame comparison is also shown. Nearly all comparisons remained significant (ANOVA p values, highlighted in green), however one change in significance is highlighted in light red.

Table S1: Secreted effector proteins with eukaryotic-like protein domains and insertion sequence (IS) coverage and counts from the genomes of *Osedax*-associated Campylobacterales epibionts.

Table S2: List of each IS family recovered from the genomes of *Osedax*-associated Campylobacterales epibionts, and functional genes carried on transposable elements shared between the epibionts listed below.

Table S3: Genes encoding attachment and secretion system proteins identified in the genomes of *Osedax*-associated Campylobacterales epibionts (bold text) compared to free-living relatives.

Table S4: Mu-like bacteriophage protein coding sequences within the genomes of the *Osedax*-associated Campylobacterales epibionts. Out of 13 ORFs recovered, 7 code for known viral proteins, and 6 are hypothetical. Amino acid sequences and accession numbers (for known proteins only) are provided.

Table S5: List of genes encoding secreted effector proteins with eukaryotic-like protein domains within the genomes of the *Osedax*-associated Campylobacterales epibionts.

Figure S1

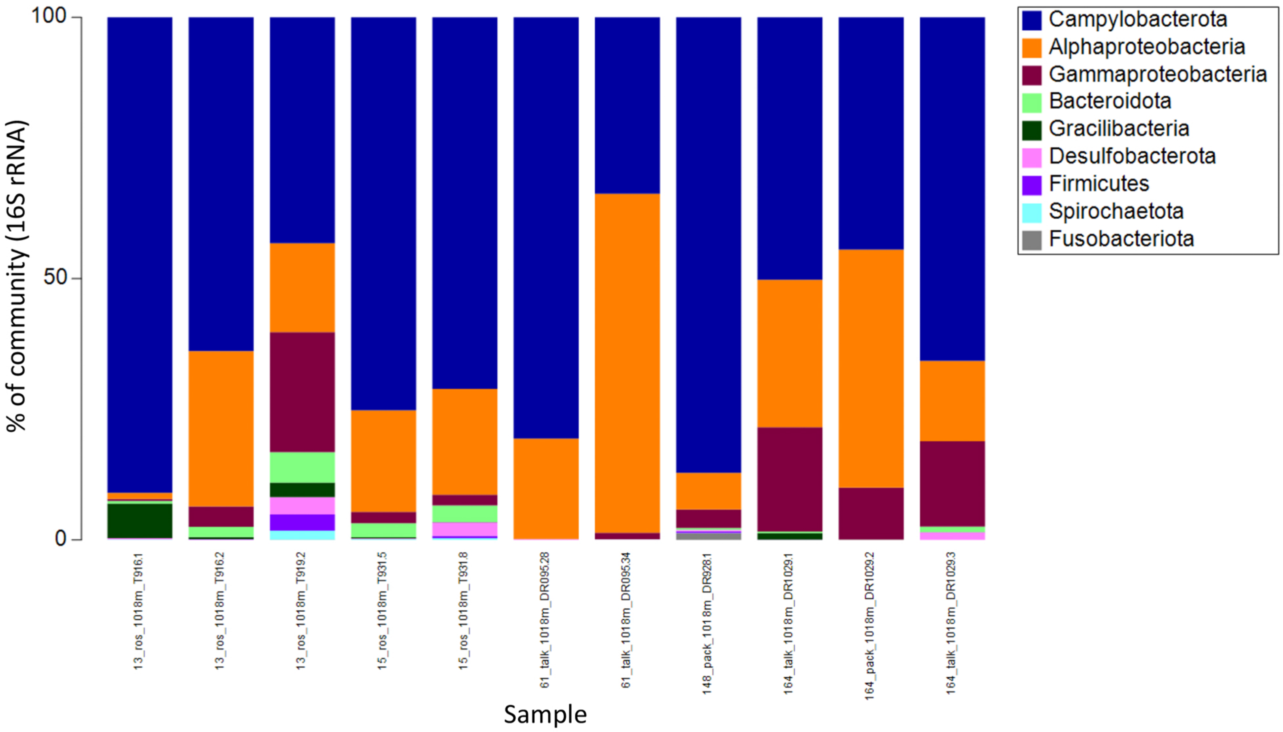

100
50
0
% of community (16S rRNA)
13_ros_1018m_T916.1
13_ros_1018m_T916.2
13_ros_1018m_T919.2
15_ros_1018m_T931.5
15_ros_1018m_T931.8
61_talk_1018m_DR095.28
61_talk_1018m_DR095.34
148_pack_1018m_DR928.1
164_talk_1018m_DR1029.1
164_pack_1018m_DR1029.2
164_talk_1018m_DR1029.3
Sample
Campylobacterota
Alphaproteobacteria
Gammaproteobacteria
Bacteroidota
Gracilibacteria
Desulfobacterota
Firmicutes
Spirochaetota
Fusobacteriota

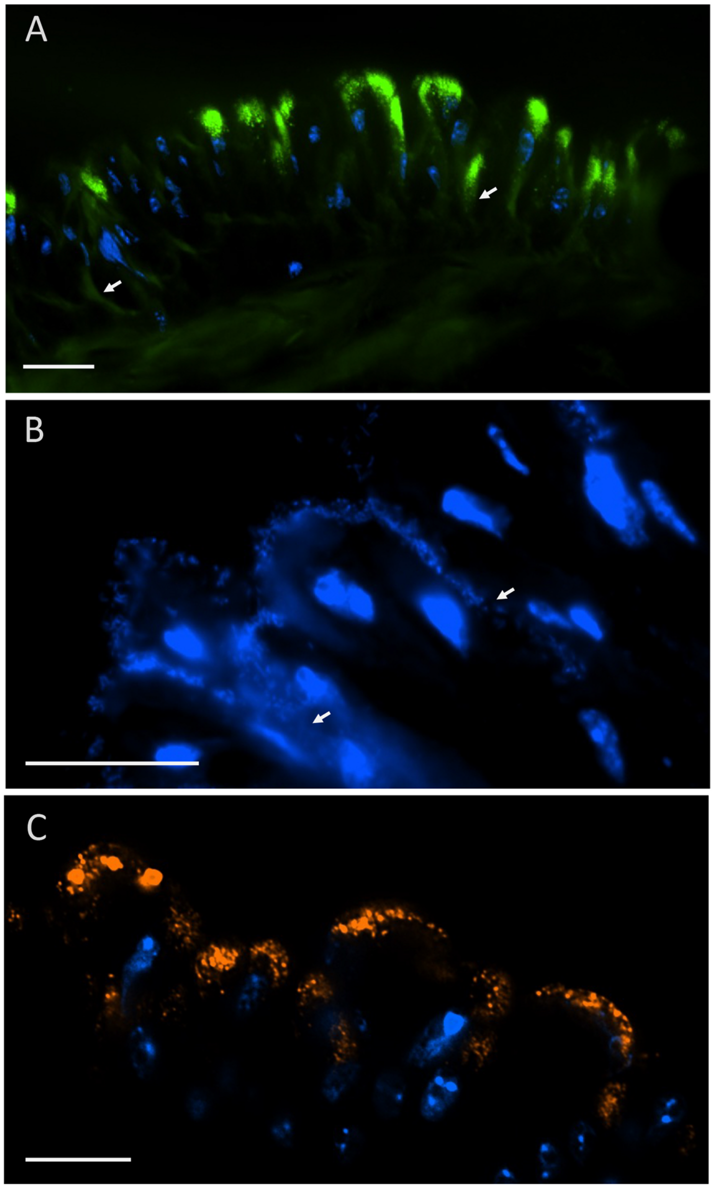
A
B
C

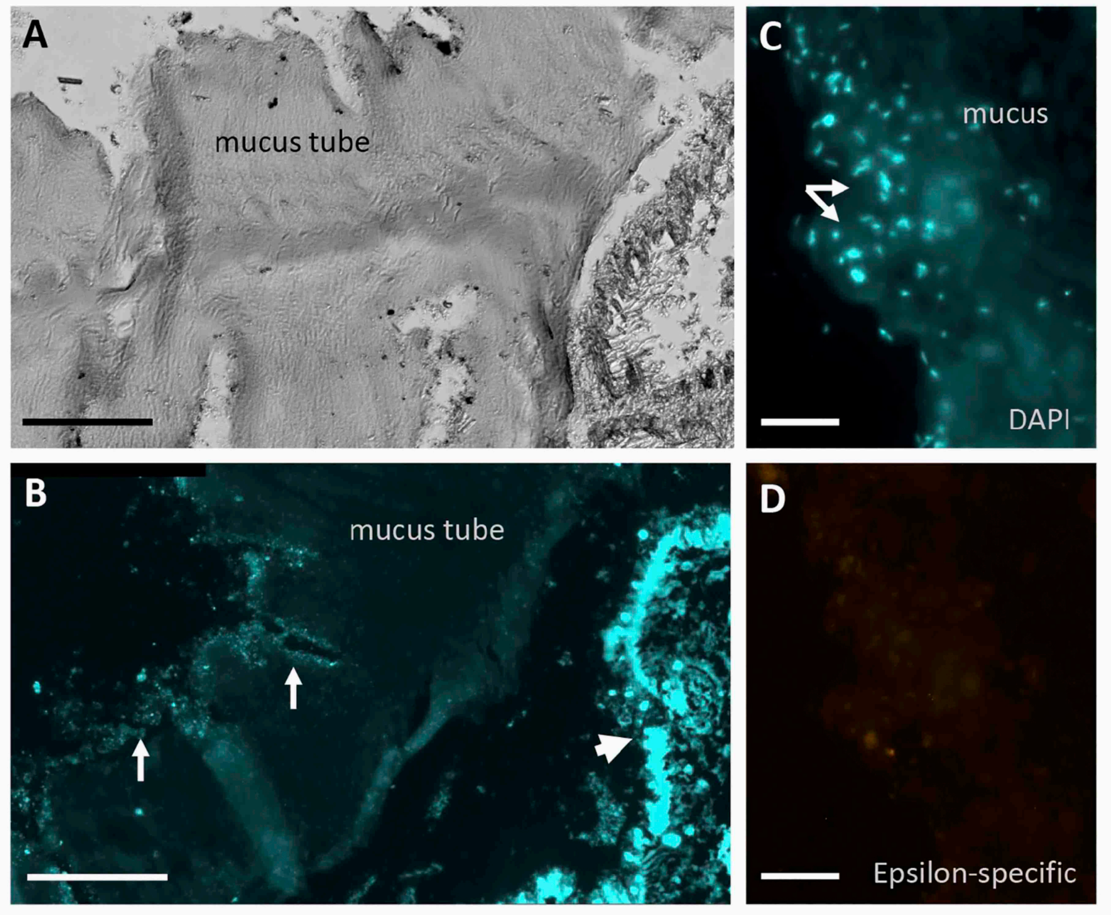
A
mucus tube
B
mucus tube
C
mucus
DAPI
D
Epsilon-specific

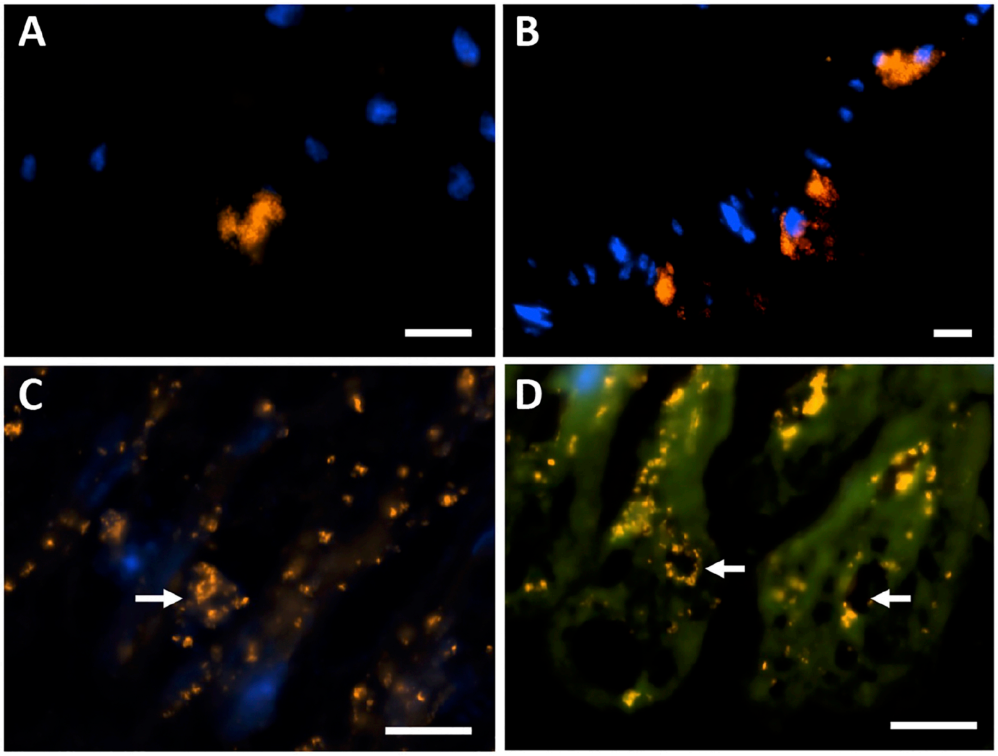
A
B
C
D

Figure S5

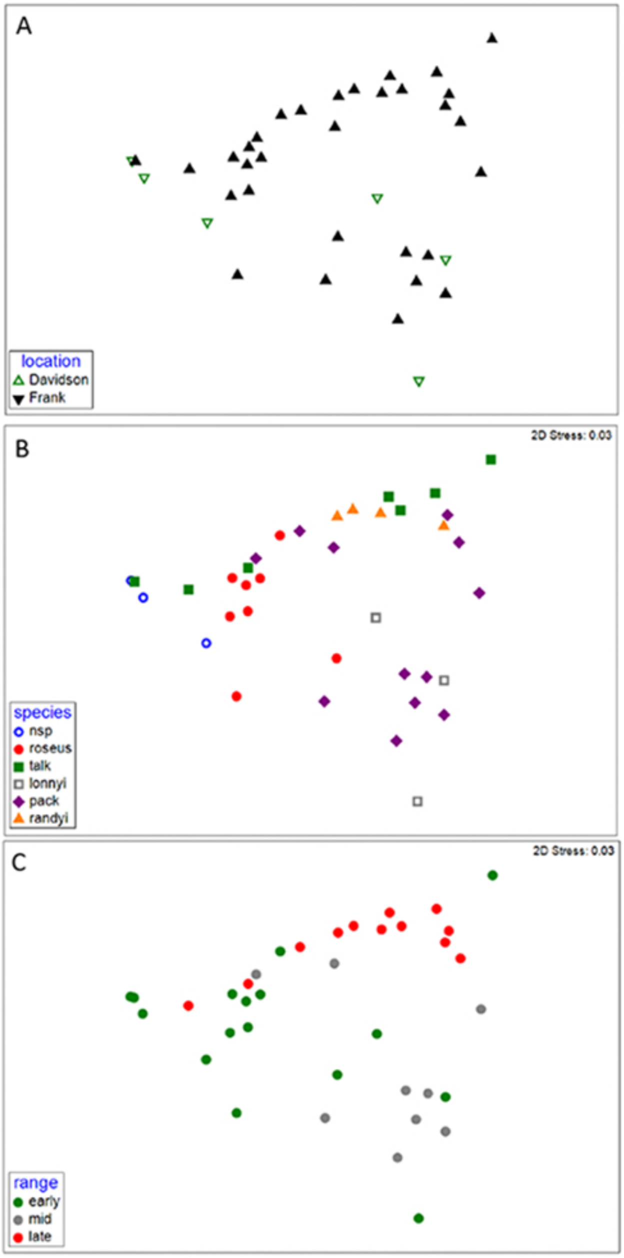
A
location
Davidson
Frank
B
2D Stress: 0.03
species
nsp
roseus
talk
lonnyi
pack
randyi
C
2D Stress: 0.03
range
early
mid
late

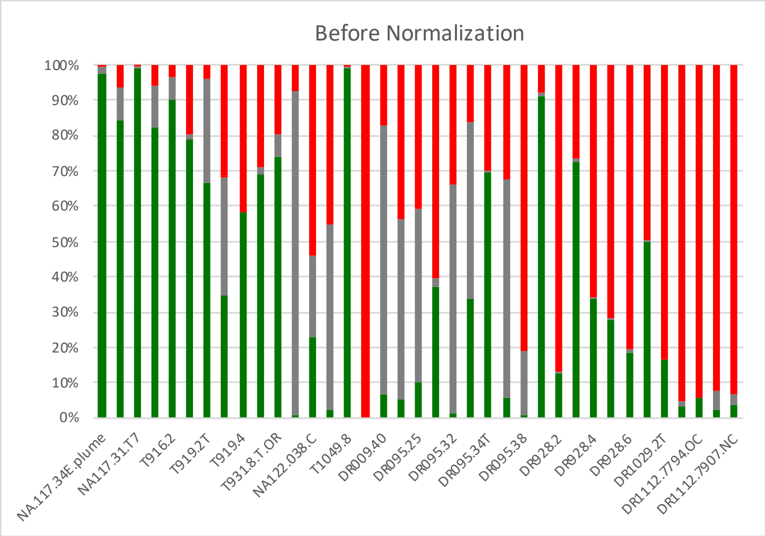

Before Normalizing

	**Arcobacter**	
	**p**	f-ratio
early v mid	0.001489	14.29991
early v late	0.008891	8.39932
mid v late	0.440609	0.62042

	**Sufurospillium**	
	**p**	f-ratio
early v mid	0.003025	11.93698
early v late	0.067747	3.72977
mid v late	0.000064	25.99343

	**Sulfurimonas**	
	**p**	f-ratio
early v mid	0.339435	0.96611
early v late	0.002332	12.14822
mid v late	0.0123	7.63738

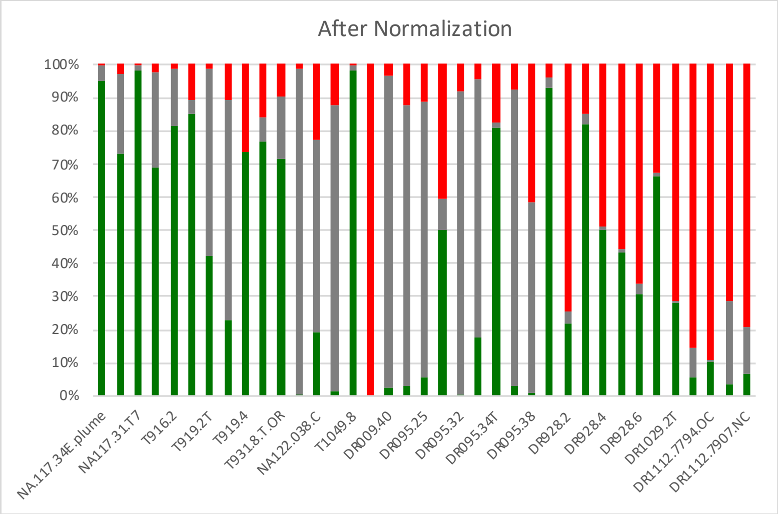

After Normalizing

	**Arcobacter**	
	**p**	f-ratio
early v mid	0.004877	10.45906
early v late	0.067417	3.73955
mid v late	0.171046	2.02397

	**Sufurospillium**	
	**p**	f-ratio
early v mid	0.004245	10.87931
early v late	0.057543	4.06033
mid v late	0.000016	32.7313

	**Sulfurimonas**	
	**p**	f-ratio
early v mid	0.892227	0.01892
early v late	0.003976	10.5872
mid v late	0.000643	16.62

Table S1	AAI vs epibiont	# secreted proteins	# with ELP	# distinct ELPs	% with ELP	# ISS	length of ISS (bp)	Genome size (bp)	% ISS Genome
***Arcobacter* relatives**									
***Arcobacter* _epibiont**	**100**	**349**	**207**	**23**	**59.3**	**227**	**130437**	**2901687**	**4.5**
CP000361_0	N/A	146	4	3	2.7	3	8472	2341251	0.4
CP001999_0	N/A	212	16	8	7.5	3	9747	3192235	0.3
CP031217_0	57.1	145	14	8	9.7	2	10197	2684688	0.4
CP031218_0	N/A	168	20	10	11.9	5	9597	2812536	0.3
CP031219_0	N/A	179	10	7	5.6	3	10491	2867150	0.4
CP031367_0	55.96	132	22	8	16.7	33	30120	1915613	1.6
CP032097_0	N/A	196	11	8	5.6	38	47115	2799949	1.7
CP032098_0	N/A	212	25	7	11.8	8	13779	2800582	0.5
CP032099_0	N/A	136	8	5	5.9	3	11241	1969846	0.6
CP032100_0	N/A	172	7	6	4.1	13	14751	2639269	0.6
CP032101_0	N/A	206	41	12	19.9	5	9837	2917098	0.3
CP032823_0	N/A	142	16	8	11.3	4	15288	2006909	0.8
CP032825_0	N/A	138	9	6	6.5	7	16098	2055914	0.8
CP035926_0	N/A	136	10	6	7.4	14	26619	1915080	1.4
CP035928_0	N/A	205	34	12	16.6	89	104376	2657991	3.9
CP036246_0	55.91	164	27	10	16.5	57	69957	2018219	3.5
CP041070_0	57.3	159	21	10	13.2	10	17334	3016922	0.6
CP042652_0	N/A	256	94	21	36.7	105	125898	3019071	4.2
CP042812_0	N/A	175	14	9	8.0	8	15111	2829476	0.5
CP043857_0	56.37	161	42	9	26.1	75	89943	2105974	4.3
GCA_000585115.1	56.44	185	12	7	6.5	7	18309	2287768	0.8
GCA_000585155.1	N/A	178	23	8	12.9	27	32601	2496885	1.3
GCA_003049765.1	N/A	211	27	11	12.8	3	9453	3502068	0.3
GCA_004023405.1	57.05	222	17	11	7.7	12	23358	3277178	0.7
GCA_004115775.1	N/A	194	14	10	7.2	6	14790	2938417	0.5
GCA_004115795.1	N/A	172	7	6	4.1	8	19677	2461253	0.8
GCA_004115805.1	N/A	191	15	8	7.9	9	18918	2775030	0.7
GCA_004116565.1	N/A	194	31	13	16.0	7	17535	3147783	0.6
Sulfurospirillum sp.									
***Sulfurospirillum* _epibiont**	**100**	**313**	**130**	**16**	**41.5**	**205**	**178377**	**2726073**	**6.5**
GCA_000024885.1	55.44	97	31	15	32.0	35	38232	2306351	1.7
GCA_000265295.1	55.15	90	21	14	23.3	13	22206	2510109	0.9
GCA_000568815.1	N/A	204	98	29	48.0	73	74763	3175729	2.4
GCA_001548035.1	N/A	129	39	17	30.2	26	21246	2606563	0.8
GCA_001548055.1	N/A	94	20	15	21.3	20	22755	2698323	0.8
GCA_001723605.1	N/A	164	62	20	37.8	19	20004	3029840	0.7
GCA_002162315.1	55.05	181	73	21	40.3	9	9756	2876536	0.3
GCA_002205395.1	55.08	176	73	21	41.5	8	10998	2876607	0.4
GCA_002309535.1	N/A	148	52	19	35.1	20	19296	2814086	0.7
GCA_008083195.1	N/A	202	96	29	47.5	67	74091	3181530	2.3
GCA_011769965.2	N/A	190	94	19	49.5	76	46296	2723916	1.7
GCA_011769985.3	55.06	162	68	17	42.0	54	38553	2777717	1.4
Sulfurimonas sp.									
***Sulfurimonas* _HC_epibiont**	**100**	**255**	**118**	**22**	**46.3**	**68**	**69864**	**2827517**	**2.5**
***Sulfurimonas* _LC_epibiont**	**88.93**	**196**	**60**	**23**	**30.6**	**41**	**57255**	**2607188**	**2.2**
CP000153.1	N/A	112	15	7	13.4	8	19320	2201561	0.9
CP002205.1	N/A	112	23	11	20.5	11	23496	2153198	1.1
CP041165_0	68.52	99	19	12	19.2	1	5079	2261034	0.2
CP041166_0	N/A	91	16	10	17.6	5	13341	1916242	0.7
CP041235_0	69.97	154	60	16	39.0	76	66732	2320257	2.9
CP041406_0	N/A	104	21	13	20.2	7	13215	2029060	0.7
CP043617_0	70.07	130	21	10	16.2	2	6423	2378486	0.3
GCA_000242915.2	N/A	321	199	28	62.0	24	19836	2952682	0.7
GCA_000445475.1	N/A	106	18	11	17.0	9	12801	2302023	0.6
GCA_009192995.1	78.46	106	15	11	14.2	6	16086	2093483	0.8

	Arcobacter	*Sulfurospirillum*	*Sulfurimonas* (HC)	*Sulfurimonas* (LC)
**IS Family**	**Total ISs**			
IS110	2	3	2	
IS1182	17	28	3	10
IS1595_ssgr_IS1016	1		1	1
IS1595_ssgr_ISNha5	2			
IS256	15		3	
IS200_IS605_ssgr_IS200		11		
IS256_ssgr_IS1249	4	8	8	
IS3_ssgr_IS150	32	12	28	22
IS3_ssgr_IS3			1	
IS3_ssgr_IS51			2	
IS3_ssgr_IS407	2			
IS30	7			3
IS4_ssgr_IS4	2	1	1	
IS4_ssgr_ISPepr1	2	14		
IS481	10	20	1	
IS5_ssgr_IS5	1	37	12	
IS5_ssgr_ISL2	96		1	
IS630	2	7		
IS66_ssgr_ISBst12		1		
IS701_ssgr_ISAba11	11			
IS91		4		
ISAs1	16	4		
ISKra4_ssgr_ISAzba1	1			
ISL3	4	11	5	5
ISNCY_ssgr_ISMae2		26		
IS21		1		
Undefined_IS_family		14		
Tn3		3		

	Flagellum*	Tad	T1SS			T5aSS	T5bSS	T6SSi													
	vairous	rcpA	omf	mfp	abc	T5aSS_P	T5bSS	tssA	evpJ	tssB	tssC	tssD	tssE	tssF	tssG	tssH	tssI	tssJ	tssK	tssL	tssM
Arcobacter																					
Arcobacter _epibiont	1		5	1	3	5	2														
CP032097_0	1		8	3	5	1															
CP031367_0	1		4	1	3																
CP031219_0	1		7	3	7																
CP031218_0	1		6	2	6	2	2	1	0	1	0	1	1	1	1	2	2	1	1	1	1
CP031217_0	1	1	7	3	7	1	1														
CP001999_0	1		12	5	9	1	4	1	0	1	0	1	1	1	1	2	2	1	1	1	1
CP000361_0	1		6	1	7		1														
GCA_004116565	1		7	3	9		1														
GCA_004115805	1		8	2	4	1															
GCA_004115795	1		8	2	4	1	2														
GCA_004115775	1		9	3	5	1	2														
GCA_004023405	1		8	1	7	1	1														
GCA_003049765	1	1	10	4	6	1	2														
GCA_000585155	1		6	2	6	2	1	1	0	1	0	1	1	1	1	2	2	1	1	1	1
GCA_000585115	1		5	1	6		2														
CP043857_0	1		5	1	3	1															
CP042812_0	1		7	2	6			1	0	1	0	1	1	1	1	2	2	1	1	1	1
CP042652_0			9	1	3																
CP041070_0	1		6	5	6			1	0	1	0	1	1	1	1	2	2	1	1	1	1
CP036246_0	1		4		2		1														
CP035928_0	1		9	3	5																
CP035926_0	1		4		2		1														
CP032825_0	1		4	1	3	2	2														
CP032823_0	1		4	2	5	1	3														
CP032101_0	1		6	2	8	1		1	0	1	0	1	1	1	1	2	2	1	1	1	1
CP032100_0	1		8	2	5	2															
CP032099_0	1		4		4		1	1	0	1	0	1	1	1	1	2	2	1	1	1	1
CP032098_0	1		6	2	4		1	1	0	1	0	1	1	1	1	2	4	1	1	1	1
Sulfurospirillum																					
Sulfurospirillum _epibiont	1	1	4	3	4																
GCA_002162315.1	1		11	2	4		1														
GCA_001723605.1	1		11	4	8	1	1														
GCA_001548055.1	1		9	4	4	1															
GCA_001548035.1	1		9	3	4		2														
GCA_000568815.1	1		8	1	3	1															
GCA_000265295.1	1		6		5	2	2														
GCA_000024885.1	1		7	2	6		1														
GCA_011769985.3	1		11	2	4																
GCA_011769965.2	1		10	2	4																
GCA_008083195.1	1		8	1	3	1															
GCA_002309535.1	1		13	2	4		1														
GCA_002205395.1	1		11	2	4		1														
Sulfurimonas																					
Sulfurimonas _HC_epibiont	1		5	4	8	2	1	1	1	1	1	15	1	1	1	2	21	1	1	1	1
Sulfurimonas _LC_epibiont	1		6	2	6		2														
CP041166_0			5	1	2																
CP041165_0	1	1	3	2	3																
CP002205.1	1	1	2		2																
CP000153.1	1		5	2	4		3														
GCA_009192995.1	1		5	2	4																
GCA_000445475.1	1		5	3	4		1														
GCA_000242915.2	1		5	2	5																
CP043617_0	1		3	1	3			1	0	1	0	1	1	1	1	2	2	1	1	1	1
CP041406_0	1		6	3	5																
CP041235_0	1	1	2	2	2																

* sctJ,sctS,sctN,flgB,sctQ,sctT,sctU,fliE,sctR,flgC,sctV

Mu-like phage proteins			
#	Annotation	Accession Number	Sequence
1	Salmonella phage hypothetical protein	NC_021774	MLRKRKMNSELFDCFTGRILGELYESFPVKKVLDIRSFDTEPCVEFYKTSPHDMEEILKGTIEFLEENELITFDRSKNGWQGGFFSGATLSLKGLQLLKKAPKSIEKDYESIGDKLIKAVKEQGIHRASTIVNEKLWGLL
2	Hypothetical bacterial protein		MSKEAILTDKYWDEYASYTYEVGTGKKKEDTLHKEQGVLFK
3	Hypothetical bacterial protein		MSLLTPNTGDEQMDKAYVHMQNHIDRCKDFETFNQHFKYVAKTDDLPKLNLWLLKVLFVQMRQVHPFDELLNHVEASKKQELESWLENMLTKVKGA
4	Yersin_phiR1_37 phage K1E myramoyl peptidase	NC_016163	MSIESPFFERDEFTCECGCGFDVADSELLDVLEGLRYEFDKPVIITGGNRCVAHNEKVQKKDNKNYKPLTSKSQHIFGKAVDFKIKDIHEDKVARYLEKEYPNTYGIGRYNGRTHIDVRENKVRWDKR
5	Ralsto_RS138 phage capsid protein	NC_029107	MAQIDDKSGHWLNKKNEGTHPDLIRADEKIKDELVEKLIKQAIELSATLNVFKKDAFEQTNDYFELLLQNYDLDAKKNSKKGNITLENYSGTMKVQIANADSISFDEKLQIAKLKIDECLHELTEGASPEIKTLITKSFEVDKKGEINAKKILALKAYDISHPKWREAMIIIDESIEIVGSKAYIRFYTRDRVDKEYKLISLDLAGA
6	Hypothetical bacterial protein		MKKDEPLKGNASELLKSIKATATFKSTYEKDISLLTAMLQNRFNKPTLDELSDYQLEELGKYLKKLDSYQTGKTEMATKNQISLIKILWSQSYKGKDTELEKALDAYIKGLGALTKKDAQKIIGGLRNGTN
7	Hypothetical bacterial protein		MTEKQRNYQSALIKSIHSSELYKDVYSDDRELYEAMLENTFGVLSSKKLSIEELINLNNFMNKKGELRVAPKKVGSTTNQINFIKTLWSKNSRNKDLNSLLTQVKKVIKRDINALESLTKDEAGKVIASIKNIKPEPKLKLNPSNNTNYKSS
8	Ralsto_RS138 phage hypothetical protein	NC_029107	MITKEQWASIKTELSAIYSGVAFTLNDKKIDVHKVQISSTKLAWIVYIDGKINLDWGHSNKKNYDPLMEQLWHKKTRSLYKESEKKKIKKIWGVRRAKKEFPELEKKYSWYKPYFNSLSVLEKQFKKLDGLELIIKDENN
9	Haemop_SuMu phage transposase	NC_019455	MTREDFKKFLDDKNLKPSRVAKSIGVSASMISQWQSNTYKGDNQSLEKKLESFKNNYDFKQSAKTSAFELKETTDLQGAFFVMEEAIVDNEMAILYGAAGTGKTTTIKEFAESRSDTRLLELVPGISTKKFLELICEKINISPEVGVDTNILNIAKELGRGDIAIIIDEAEHLTVRSLEAIRRVWDFTPFALILVGTPVLIKNLKGNSGELLQLYSRPSGKYEFKGLRANEFELFFGDFGKEVKKYTTHMRRAVSLYRKATRFALMNNEKLDVKHIKLASTMVFLD
10	Ralsto_RS138 phage integrase	NC_029107	MNSKEVASLVGVSYKSGIVQRITKKALENSSCTMVIKGLTFGFELVHGKAYLYTEFKAKKKTQKHINKVAISSLDELDSFDITASKHSQEDKVLIIAFYKKYNYSLSAIIKSLYAKSFLAFDDKLIASQQRKISRWLSDFNKNGASSLEDKRGRDNAFRKIDEELLIKSIWGTGSQGVRNNYYGAYDMYCGMVQSQNGLMYDWSEKAKEKIISYNAYVNAYKKVVKSDPQLKGYLTGGIDALLQDYPVGIKDIAYPNQEWQVDATVFDFMCKIPDETKEDGYRVGRVHMTAVKDTFTKQAVANLVETIDSYSQVRVLYKAFQKMGIPENIYTDNGMDYVSNHYQKLLIDIGICQIRAVAGQGRQKGSIERYFGVVQGRWNKIPGYIGGDVSKRKKIEDQYASHLGVKTSKATRIETHKLLTLDELRLVVDNLLDMQYESYKAHQEFIASTNMMSDIYSKLGKYGTRTLNTEGIRYNNMTFQGSSLWEAHLNKGEVVTFYENIDNVNELYIYKQNQYIGVVKNKELGAECMNQEDHKKVTKIYKQTNIKPKLKILDEANEELEKQKVAFISAVSGHKPLYTTQSDMSPKTSKEANNPVTAPISLTSKQDSELIKFIRSVS
11	Hypothetical bacterial protein		MRELLVAKLKKARQNYIVIYTTNHTQARTLALHCAGRECMEFRLKLHNLDVGNE
12	Hypothetical bacterial protein		MATLELSKKIKKDYVTVENFCTKHNIKYNTYKGVVNGSHPSQPIVKILIKEGYIKSADDLLKKAA
13	Tenaci_pT24 phage tape measure chaperone	NC_049383	MSKEKKLFGLCQSQDLNVSLTWQKINDWSVEIYLGNTYRKIFYTDGHTRKKDALKEAIEFMETFDRMKWQSEPQEITIGLKVSIKQPTVIDTVKLFIDHLDKHHDDGDKRYEILAMCAASQQWILKNP

Table S5

Arcobacter

Pfam ID	Annotation
PF00133	tRNA synthetases class I (I, L, M and V)
PF00145	C-5 cytosine-specific DNA methylase
PF00665	Integrase core domain
PF00872	Transposase, Mutator family
PF01527	Transposase
PF01880	Desulfoferrodoxin
PF02460	Patched family
PF02516	Oligosaccharyl transferase STT3 subunit
PF02689	Helicase
PF02807	ATP:guanido phosphotransferase, N-terminal domain
PF03190	Protein of unknown function, DUF255
PF10609	NUBPL iron-transfer P-loop NTPase
PF11074	Domain of unknown function(DUF2779)
PF12344	Ultra-violet resistance protein B
PF13090	Polyphosphate kinase C-terminal domain 2
PF13276	HTH-like domain
PF13359	DDE superfamily endonuclease
PF13384	Homeodomain-like domain
PF13603	Leucyl-tRNA synthetase, Domain 2
PF13613	Helix-turn-helix of DDE superfamily endonuclease
PF13683	Integrase core domain
PF14336	Domain of unknown function (DUF4392)
PF14622	Ribonuclease-III-like

***Sulfurimonas* (HC)**

Pfam ID	Annotation
PF00145	C-5 cytosine-specific DNA methylase
PF00665	Integrase core domain
PF01142	tRNA pseudouridine synthase D (TruD)
PF01527	Transposase
PF01871	AMMECR1
PF01875	Memo-like protein
PF02516	Oligosaccharyl transferase STT3 subunit
PF02689	Helicase
PF02807	ATP:guanido phosphotransferase, N-terminal domain
PF02943	Ferredoxin thioredoxin reductase catalytic beta chain
PF03162	Tyrosine phosphatase family
PF03190	Protein of unknown function, DUF255
PF05598	Transposase domain (DUF772)
PF05735	Thrombospondin C-terminal region
PF08238	Sel1 repeat
PF09994	Uncharacterized alpha/beta hydrolase domain (DUF2235)
PF10520	Lipid desaturase domain
PF10563	Delta carbonic anhydrase
PF12513	Mitochondrial degradasome RNA helicase subunit C terminal
PF13181	Tetratricopeptide repeat
PF13276	HTH-like domain
PF16114	ATP citrate lyase citrate-binding

Sulfurospirillum

Pfam ID	Annotation
PF00068	Phospholipase A2
PF01142	tRNA pseudouridine synthase D (TruD)
PF01555	DNA methylase
PF02112	cAMP phosphodiesterases class-II
PF02527	rRNA small subunit methyltransferase G
PF02807	ATP:guanido phosphotransferase, N-terminal domain
PF06414	Zeta toxin
PF09834	Predicted membrane protein (DUF2061)
PF10323	Serpentine type 7TM GPCR chemoreceptor Srv
PF12513	Mitochondrial degradasome RNA helicase subunit C terminal
PF13237	4Fe-4S dicluster domain
PF13276	HTH-like domain
PF13384	Homeodomain-like domain
PF13683	Integrase core domain
PF14022	Protein of unknown function (DUF4238)
PF14743	DNA ligase OB-like domain

***Sulfurimonas* (LC)**

Pfam ID	Annotation
PF00665	Integrase core domain
PF01142	tRNA pseudouridine synthase D (TruD)
PF01527	Transposase
PF01871	AMMECR1
PF01875	Memo-like protein
PF02112	cAMP phosphodiesterases class-II
PF02516	Oligosaccharyl transferase STT3 subunit
PF02689	Helicase
PF02807	ATP:guanido phosphotransferase, N-terminal domain
PF02943	Ferredoxin thioredoxin reductase catalytic beta chain
PF03162	Tyrosine phosphatase family
PF03486	HI0933-like protein
PF05203	Hom_end-associated Hint
PF05204	Homing endonuclease
PF10563	Delta carbonic anhydrase
PF11074	Domain of unknown function(DUF2779)
PF12513	Mitochondrial degradasome RNA helicase subunit C terminal
PF12588	Phophatidylserine decarboxylase
PF13276	HTH-like domain
PF13332	Hemagglutinin repeat
PF13455	Meiotically up-regulated gene 113
PF13597	Anaerobic ribonucleoside-triphosphate reductase
PF16114	ATP citrate lyase citrate-binding
